# CD44 signaling in Müller cells impacts photoreceptor function and survival in healthy and diseased retinas

**DOI:** 10.1186/s12974-024-03175-8

**Published:** 2024-08-02

**Authors:** Monika Ayten, Tobias Straub, Lew Kaplan, Stefanie M. Hauck, Antje Grosche, Susanne F. Koch

**Affiliations:** 1 Department of Pharmacy, Center for Drug Research, Ludwig-Maximilians-Universität München, Munich, 81377 Germany; 2 Bioinformatics Unit, Biomedical Center Munich, Ludwig-Maximilians-Universität München, Planegg-Martinsried, 82152 Germany; 3 Department of Physiological Genomics, Biomedical Center Munich, Ludwig-Maximilians-Universität München, Planegg-Martinsried, 82152 Germany; 4 Metabolomics and Proteomics Core, Helmholtz Zentrum München, German Research Center for Environmental Health, Neuherberg, Germany

**Keywords:** CD44, Glutamate, Retinitis pigmentosa, SLC1A2, Müller cells, Gliosis, Inflammation

## Abstract

Retinitis pigmentosa (RP), an inherited retinal disease, affects 1,5 million people worldwide. The initial mutation-driven photoreceptor degeneration leads to chronic inflammation, characterized by Müller cell activation and upregulation of CD44. CD44 is a cell surface transmembrane glycoprotein and the primary receptor for hyaluronic acid. It is involved in many pathological processes, but little is known about CD44’s retinal functions. CD44 expression is also increased in Müller cells from our *Pde6b*^*STOP/STOP*^ RP mouse model. To gain a more detailed understanding of CD44’s role in healthy and diseased retinas, we analyzed *Cd44*^*−/−*^ and *Cd44*^*−/−*^*Pde6b*^*STOP/STOP*^ mice, respectively. The loss of CD44 led to enhanced photoreceptor degeneration, reduced retinal function, and increased inflammatory response. To understand the underlying mechanism, we performed proteomic analysis on isolated Müller cells from *Cd44*^*−/−*^ and *Cd44*^*−/−*^*Pde6b*^*STOP/STOP*^ retinas and identified a significant downregulation of glutamate transporter 1 (SLC1A2). This downregulation was accompanied by higher glutamate levels, suggesting impaired glutamate homeostasis. These novel findings indicate that CD44 stimulates glutamate uptake via SLC1A2 in Müller cells, which in turn, supports photoreceptor survival and function.

**Supplementary Information:**

The online version contains supplementary material available at 10.1186/s12974-024-03175-8.

## Introduction

Müller cells, the main support cells in the retina, span the entire thickness of the tissue and envelop all retinal neurons. Thus, Müller cells are positioned to fulfill multiple functions essential for neuronal well-being [1]. For example, they provide metabolic support to photoreceptors, regulate retinal blood flow, and they contribute to neuronal signaling by uptake and recycling of glutamate [2–4]. In addition, Müller cells express attachment points for the interphotoreceptor matrix, a highly organized structure between the photoreceptors and the retinal pigment epithelium (RPE) [5]. An important attachment point is the hyaluronic acid (HA)-binding motif CD44, a transmembrane glycoprotein localized in the microvilli of Müller cells [5–9].

CD44 is found in a variety of tissues, including the central nervous system, lung, epidermis, liver, pancreas, hematopoietic and lymphatic system [10, 11]; in the lymphatic system, CD44 has been shown to play crucial roles in T-cell differentiation in the thymus [12] and migration of lymphocytes to lymph nodes [13, 14]. CD44 is also involved in many pathological processes such as inflammation, immune responses, wound healing, cancer progression, and epigenetic plasticity in tumors [15, 16]. Despite the many roles described to CD44, little is known about its function in the healthy and diseased retina.

In retinitis pigmentosa (RP), CD44 was found to be dramatically upregulated [17]. For example, in the retinal degeneration slow (rds) mouse [18] and in the Royal College of Surgeons (RCS) rat model [19], CD44 was increased in Müller cells. Increased CD44 expression in microglia was observed in the *Cngb1*-knockout mouse model by FACS [20]. RP is a group of retinal degenerative diseases manifesting in adolescence or early adulthood, and the most common cause of inherited blindness [21–23]. Patients with RP initially experience night blindness, followed by a gradual narrowing of the visual field and, ultimately, loss of daylight vision. This progressive loss of vision occurs in parallel with the progressive loss of photoreceptor cells. The photoreceptor degeneration activates Müller cells – a process, known as Müller cell gliosis, marked by upregulation of glial fibrillary acidic protein (GFAP) [24, 25].

Given the key roles played by Müller cells in healthy retina, and the changes they undergo in many retinal degenerative diseases [26–28], it is important to understand their role in RP pathogenesis. Here, we study the effect of CD44 signaling on retinal morphology in healthy and diseased retinas. Using our RP *Pde6b*^*STOP/STOP*^ mouse model, we confirmed that CD44 expression is upregulated in Müller cells. Interestingly, when we crossed our RP mice with *Cd44*^*-/-*^ mice, we discovered that retinas exhibited significantly faster photoreceptor degeneration, decreased retinal function, increased glutamate levels, and, in Müller cells, decreased expression of glutamate transporter Slc1a2. This work suggests a role of CD44 in glutamate homeostasis and emphasizes the significance of CD44 for retinal integrity and function, both in healthy and diseased conditions.

## Results

### Upregulation of CD44 in Müller cells from RP mouse models

**Fig. 1:**
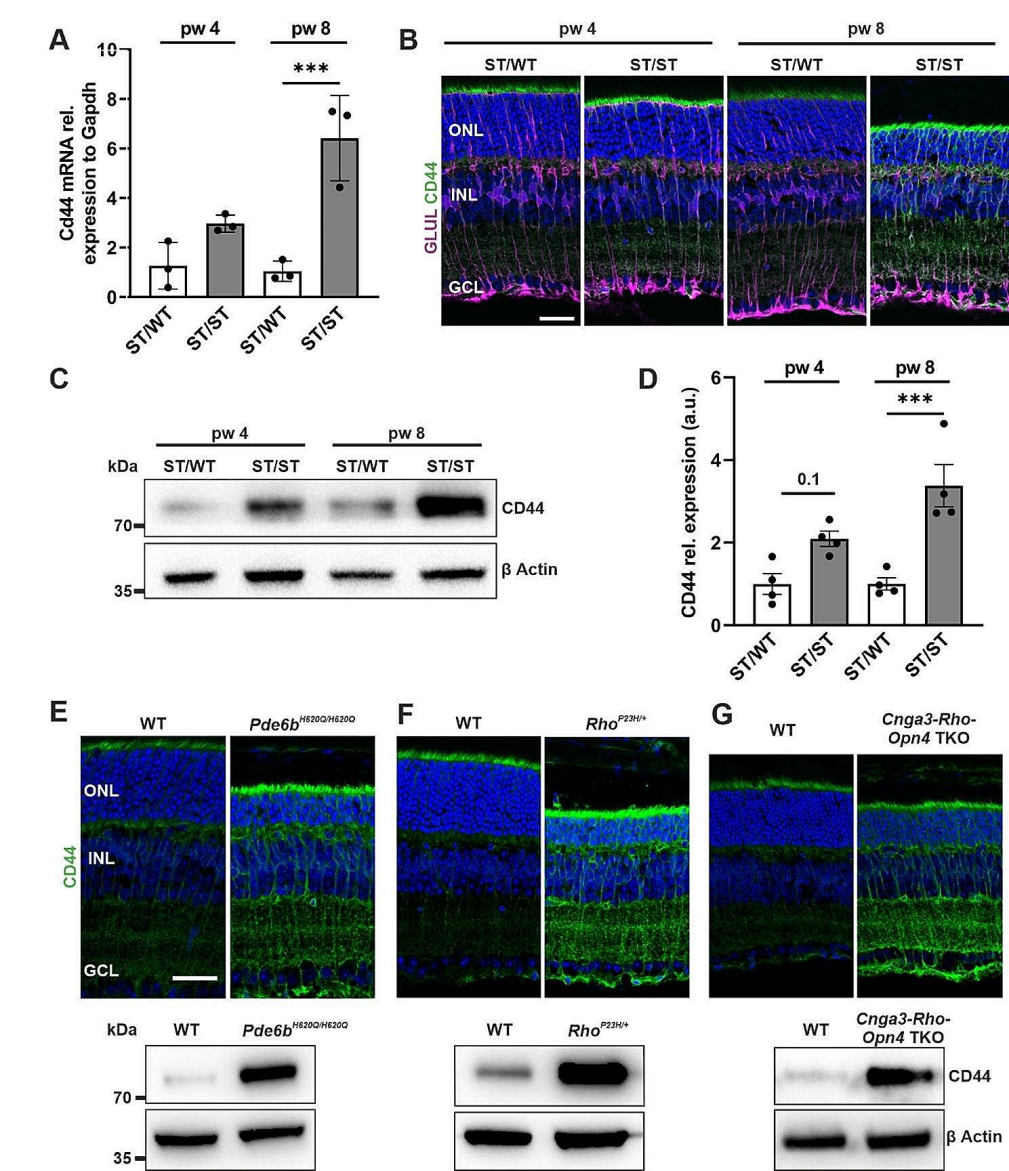
Progressive photoreceptor degeneration results in upregulation of CD44 expression in Müller cells. (**A-D**) Retinas from *Pde6b*^*STOP/WT*^ (ST/WT) and *Pde6b*^*STOP/STOP*^ (ST/ST) mice were analyzed at 4 and 8 weeks of age. Expression of CD44 increases with disease progression. **(A)** Quantitative analysis of *Cd44* mRNA by qRT-PCR. *N* = 3 per group. (**B**) Representative images of retinal sections immunostained for glutamine synthetase (GLUL) to visualize Müller cells and for CD44. CD44 is mainly expressed in the apical microvilli of Müller cells. Scale bar, 30 μm. (**C**) Representative CD44 immunoblot of retinal lysates. β-Actin was used as a loading control. (**D**) Quantitative analysis of CD44 immunoblots. *N* = 4 per group. (**A**,** D**) Data, presented as mean ± SEM, were compared by ANOVA. *** *P ≤* .001. (**E-G**) Retinas from 3 retinal degeneration mouse models were analyzed: *Pde6b*^*H620Q/H620Q*^ (week 4), *Rho*^*P23H/+*^ (week 9) , and *Cnga3*^−/−^, Rho^−/−^, Opn4^*−/−*^ triple-knockout mice (week 5). (**Upper panel**) Representative images of retinal sections immunostained for CD44. Scale bar, 30 μm. (**Lower panel**) Representative CD44 immunoblots of retinal lysates. β-Actin was used as a loading control. ONL, outer nuclear layer; INL, inner nuclear layer; GCL, ganglion cell layer

Müller cells upregulate CD44 in response to photoreceptor degeneration in the retinal degeneration slow (*rds*) mouse [18]. To evaluate whether CD44 is also upregulated in other RP mouse models, we first analyzed CD44 expression levels in retinas from *Pde6b*^*STOP/STOP*^ (ST/ST) mice by RT-qPCR, immunohistochemistry, and immunoblot. These mice contain a floxed stop cassette in both *Pde6b* alleles that prevents PDE6B expression (Fig. S1A), leading to progressive photoreceptor degeneration [29]. To understand whether there is an association between CD44 expression and photoreceptor degeneration, we analyzed retinas at 4- and 8-weeks of age (i.e., at early- and mid-disease stage). In ST/ST retinas (vs. age-matched *Pde6b*^*STOP/WT*^ (ST/WT) control), *Cd44* mRNA was significantly upregulated at 8 weeks of age (*P* ≤ .001) (Fig. 1A). Next, we analyzed retinal sections that were co-immunostained for glutamine synthetase (GLUL) to visualize Müller cells and CD44. In line with previous studies [30, 31], CD44 was expressed in Müller cells, and particularly localized to the apical microvilli of Müller cells (Fig. 1B and S1B). Notably, in ST/ST retinas, CD44 expression was enhanced in the microvilli of Müller cells compared to ST/WT controls (Fig. 1B). The microvilli extend into the subretinal space between the inner segments of photoreceptors, but are not in contact with rod photoreceptor outer segments (Fig. S1C). To further validate CD44 upregulation in ST/ST compared to ST/WT retinas, immunoblotting was performed (Fig. 1C). The immunoblot quantification demonstrated that CD44 protein expression showed a trend of upregulation at 4 weeks (*P* = .1) and was significantly upregulated at 8 weeks (*P* ≤ .001) (Fig. 1D). These data suggest an association between CD44 expression level and photoreceptor degeneration – the highest level of CD44 was detected in 8-week-old ST/ST retinas (i.e., in the retina with the most extensive degeneration). Since CD44 is expressed in reactive microglia cells from *Cngb1*-knockout retinas [20] and from retinas following endotoxin induced uveitis [32], we analyzed retinal sections and retinal flatmounts from 12-week-old ST/ST mice that were co-immunostained for IBA1 to visualize microglia/macrophages and CD44. At 12-weeks of age, microglia cells have migrated into the ONL (**Fig. **S1D). CD44 is highly expressed in the microvilli of Müller cells but appears not to be expressed in microglia/macrophages (Fig. S1D/E). To further confirm the cell-type specific expression pattern of CD44, we analyzed previously published single-cell RNA sequencing (scRNAseq) mouse data from control and light-damaged retinas [33]. These data showed CD44 expression in Müller cells but not in other retinal cells. In line with our findings, CD44 expression was increased in light-damaged retinas (Fig. S1F).

Next, we analyzed CD44 expression in retinas from 3 other retinal degeneration mouse models by immunohistochemistry and immunoblot. We investigated another autosomal recessive RP mouse model caused by a missense mutation in the *Pde6b* gene (*Pde6b*^*H620Q/H620Q*^) [34], an autosomal dominant RP mouse model caused by a mutation in the rhodopsin gene (*Rho*^*P23H/+*^) [35], and *Cnga3*^*−/−*^, *Rho*^*−/−*^, *Opn4*^*−/−*^ triple-knockout mice [36, 37]. CD44 was highly expressed in all 3 other retinal degeneration mouse models (Fig. 1E-G), suggesting that photoreceptor degeneration, independent of the genetic cause, leads to upregulation of CD44 expression in Müller cells.

### Loss of CD44 does not affect GFAP expression levels

**Fig. 2:**
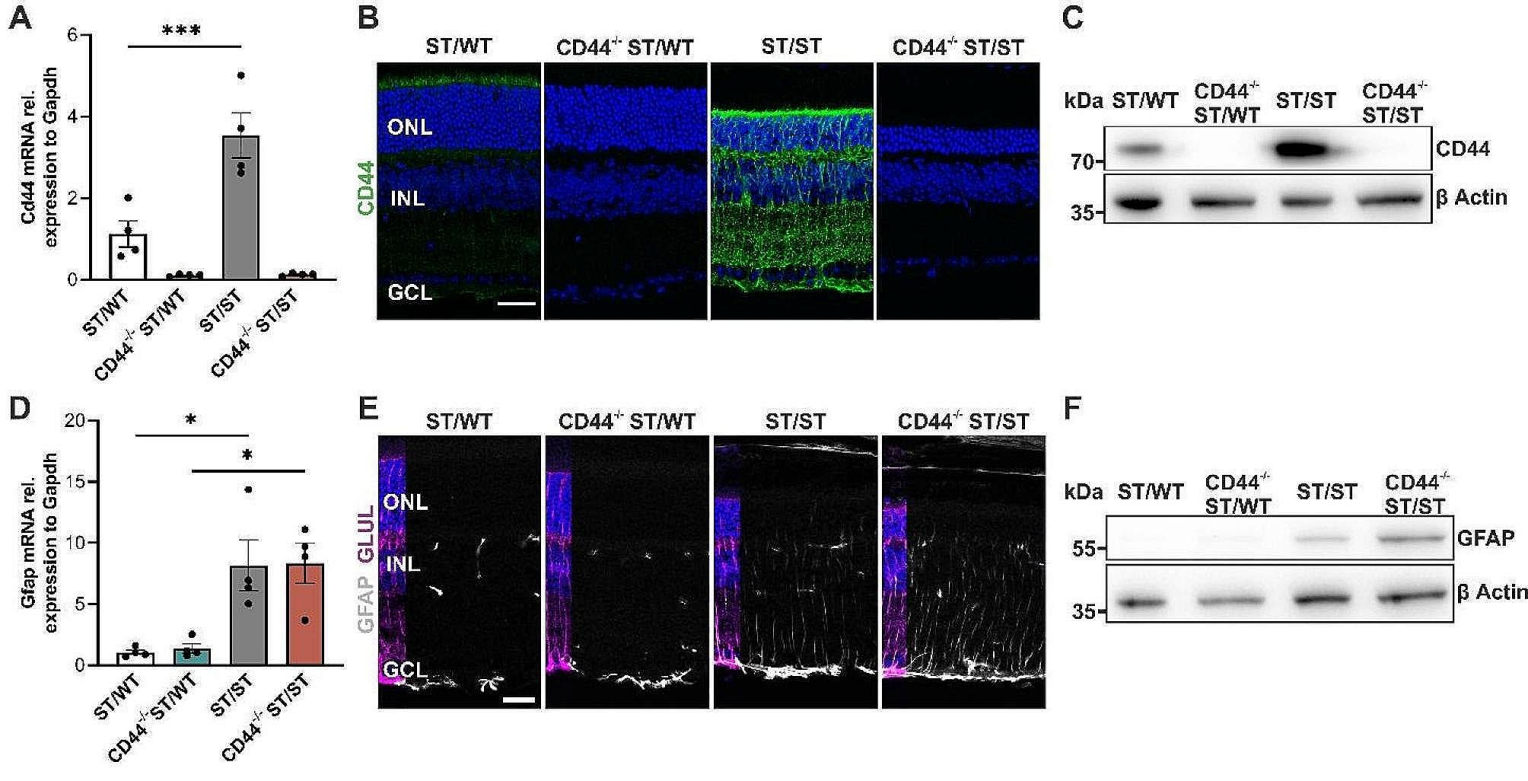
Absence of CD44 does not affect GFAP expression levels. Retinas from *Pde6b*^*STOP/WT*^ (ST/WT), *Cd44*^*−/−*^Pde6b^STOP/WT^(CD44^−/−^ ST/WT), *Pde6b*^*STOP/STOP*^ (ST/ST), and *Cd44*^*−/−*^*Pde6b*^*STOP/STOP*^ (CD44^−/−^ ST/ST) mice were analyzed at 8 weeks of age. (**A**) Quantitative analysis of *Cd44* mRNA by qRT-PCR. (**B**) Representative images of retinal sections immunostained for CD44. Scale bar, 30 μm. (**C**) Representative CD44 immunoblot of retinal lysates. β-Actin was used as a loading control. (**D**) Quantitative analysis of *Gfap* mRNA by qRT-PCR. (**A**,** D**)* N* = 4 per group. Data, presented as mean ± SEM, were compared by ANOVA. * *P* ≤ .05; *** *P* ≤ .001. (**E**) Representative images of retinal sections immunostained for GLUL and GFAP. Scale bar, 25 μm (**F**) Representative GFAP immunoblot of retinal lysates. β-Actin was used as a loading control. ONL, outer nuclear layer; INL, inner nuclear layer; GCL, ganglion cell layer

After confirmation that CD44 is expressed in Müller cells in the retina (Fig. 1B, Fig. S1B-F), we utilized a commercially available CD44 global knockout mouse line (*Cd44*^*−/−*^) and crossed it with our *Pde6b*^*STOP*^ mouse line. This crossbreeding strategy yielded *Cd44*^*−/−*^*Pde6b*^*STOP/WT*^ (CD44^−/−^ ST/WT) and *Cd44*^*/−*^*Pde6b*^*STOP/STOP*^ (CD44^−/−^ ST/ST) mice. Incorporating CD44^−/−^ ST/WT mice into our study allowed us to study the impact of CD44 loss on normal retinal morphology, while the analysis of CD44^−/−^ ST/ST mice provided insight into the role of CD44 in RP. We first confirmed that CD44 is absent in retinas from 8-week-old CD44^−/−^ ST/WT and CD44^− /−^ ST/ST mice by RT-qPCR (Fig. 2A), immunohistochemistry (Fig. 2B), and immunoblot (Fig. 2C). In line with previous experiments (Fig. 1A-D), CD44 was upregulated in ST/ST retinas compared to ST/WT on both RNA (ANOVA, *** *P* ≤ .001) and protein level (Fig. 2A-C). Since Müller cells also upregulate glial fibrillary acidic protein (GFAP) in response to photoreceptor degeneration [28], we examined GFAP expression at 8 weeks of age in CD44^−/−^ ST/WT and CD44^− /−^ST/ST mice compared to ST/WT and ST/ST mice, respectively (Fig. 2D-F). As expected, the mRNA levels of *Gfap* were upregulated in both ST/ST and CD44^/−^ ST/ST retinas (*P* ≤ .05) (vs. age-matched ST/WT and CD44^−/−^ST/WT, respectively). On the other hand, *Gfap* was neither different between ST/WT and CD44^−/−^ ST/WT mice nor between ST/ST and CD44^/−^ ST/ST mice (Fig. 2D). In retinal sections from ST/WT and CD44^−/−^ ST/WT mice, GFAP immunoreactivity was limited to the ganglion cell layer. In ST/ST and CD44^−/−^ ST/ST mice, some radially oriented stress fibers were immunolabeled (Fig. 2E). Immunoblotting confirmed increased GFAP expression in ST/ST and CD44^−/−^ ST/ST mice (Fig. 2F). These data show that loss of CD44 does not alter GFAP expression.

Since CD44 is the transmembrane receptor for hyaluronic acid (HA) [38], we next quantitatively measured the HA concentration in retinal lysates at 4 and 8 weeks of age using an HA ELISA assay (Fig.S2). Interestingly, HA was significantly upregulated in 8-week-old CD44^−/−^ ST/WT retinas compared to ST/WT, with HA concentrations measuring 95 ± 23 (SEM) ng/ml and 16 ± 1.2 ng/ml, respectively (ANOVA, *P* = .025). There were no significant differences between the two groups at 4 weeks of age, which might be due to low statistical power (low sample size and variation within the sample). The HA levels in ST/ST retinas were 89 ± 12 ng/ml and 58 ± 20 ng/ml at 4 and 8 weeks of age, respectively, and appear to be higher compared to age-matched ST/WT; however, these differences are not statistically significant (ANOVA, *P* = .2 and *P* = .3).

### Loss of CD44 contributes to photoreceptor degeneration and rod bipolar remodeling

**Fig. 3:**
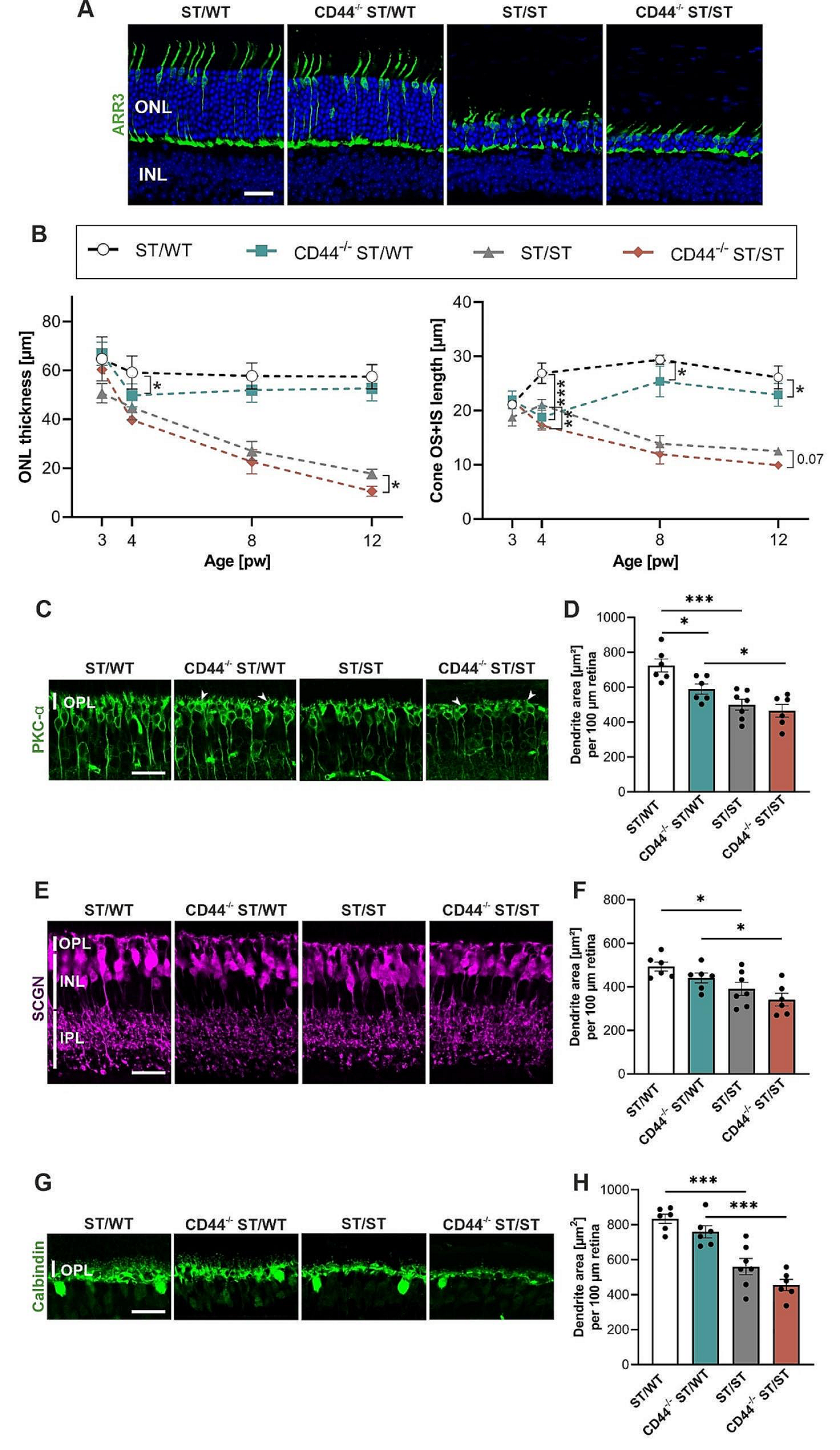
Progression of photoreceptor degeneration and inner retinal remodeling in CD44-deficient mice. Retinas from ST/WT, CD44^−/−^ ST/WT, ST/ST, and CD44^−/−^ ST/ST mice were analyzed. (**A**) Representative images of retinal sections from 12-week-old mice immunostained for ARR3 (cones). Scale bar, 25 μm. (**B**) ONL thickness and cone inner and outer segment length at 3, 4, 8, and 12 weeks of age. pw 3, *N* = 5 per group. pw 4, *N* = 6 per group. pw 8, *N* = 5 for ST/WT, and ST/ST, *N* = 7 for CD44^−/−^ ST/WT, *N* = 6 for CD44^−/−^ ST/ST. pw 12, *N* = 8 for ST/WT, *N* = 6 for CD44^/−^ ST/WT, and CD44^−/−^ ST/ST, *N* = 5 for ST/ST. (**C**,** E**,** G**) Representative images of retinal sections from 12-week-old mice immunostained for PKC–α (rod bipolar cells) (**C**), SCGN (cone bipolar cells) (**E**), and calbindin (horizontal cells) (**G**). Arrowheads indicate shorter and less-branched bipolar dendrites. Scale bars, 25 μm. (**D**,** F**,** H**) Quantification of dendritic area from rod bipolar cells (**D**), cone bipolar cells (**F**), and horizontal cells (**H**). pw 12, *N* = 6 for ST/WT, CD44^−/−^ ST/WT, and CD44^−/−^ ST/ST, *N* = 7 for ST/ST. Data, presented as mean ± SEM, were compared by ANOVA. * *P* ≤ .05; ** *P* ≤ .01; *** *P* ≤ .001. ONL, outer nuclear layer; INL, inner nuclear layer; IS, inner segment; OS, outer segment; OPL, outer plexiform layer; IPL, inner plexiform layer

To analyze the effect of CD44 on retinal morphology and disease progression, we analyzed morphological changes in photoreceptors and inner retinal neurons in retinal sections (Fig. 3). Cone arrestin (ARR3) was used to visualize cone photoreceptors (Fig. 3A). To quantify photoreceptor degeneration, outer nuclear layer (ONL) thickness was measured at 3, 4, 8, and 12 weeks of age. At 3 weeks of age, the ONL thickness of ST/WT and CD44^−/−^ ST/WT retinas were 65 μm ± 4.0 (SEM) µm and 67 μm ± 2.1 μm, respectively. The mean ONL thickness was not significantly different between the two groups, indicating that CD44 does not affect photoreceptor number before postnatal week 3 (Fig. 3B, left panel). At 4 weeks of age, ONL thickness of ST/WT retinas was slightly decreased to 59 μm ± 2.8 μm due to normal programmed cell death associated with neuronal differentiation and maturation [39, 40] (Fig. 3B, left panel). In CD44^−/−^ ST/WT mice, the ONL thickness decreased to 50 μm ± 1.9 μm (ANOVA, *P* ≤ .05; vs. age-matched ST/WT control). These data suggest an additional CD44-related photoreceptor cell death. Between weeks 4 and 12, the ONL thickness was stable in both ST/WT and CD44^−/−^ ST/WT retinas. In contrast, in 3-week-old ST/ST and CD44^−/−^ ST/ST mice, the ONL thickness was 51 μm ± 1.8 μm and 60 μm ± 2.1 μm, respectively, and progressively became thinner over time. At 12 weeks of age, the ONL thickness of ST/ST and CD44^−/−^^-^ ST/ST retinas were 17 μm ± 0.8 μm and 11 μm ± 0.8 μm, respectively (ANOVA, *P* = .03) (Fig. 3B, left panel). For a comprehensive histological examination, retinal sections from 3-, 4-, 8-, and 12-week-old mice were analyzed using hematoxylin and eosin staining (Fig. S3A). At 3 weeks of age, the retinal morphology was similar between the different groups, suggesting that photoreceptor cells develop normally. At 4, 8, and 12 weeks of age, the ONL thickness from CD44^−/−^ ST/WT retinas appeared somewhat reduced compared to age-matched ST/WT. In ST/ST and CD44^−/−^ ST/ST mice, photoreceptors degenerated over time, reflected by a progressive decrease in ONL thickness. Consistent with Fig. 3B, the ONL thickness in CD44^−/−^ ST/ST retinas appeared thinner compared to ST/ST retinas at weeks 4, 8, and 12 (**Fig. S3A**). We next quantified ONL thickness at multiple positions across a 2000-µm length of the central retina at 3 and 8 weeks of age for all groups (Fig. S3B). At 3 weeks of age, no significant differences were observed between the groups. In line with Fig. 3B, at 8 weeks of age, the ONL thickness was reduced in ST/ST and CD44^−/−^ST/ST compared to ST/WT and CD44^−/−^ ST/WT, respectively.

We next analyzed the effect of CD44 loss on cone outer segments. At 3 weeks of age, the cone inner segment (IS) and outer segment (OS) length of ST/WT and CD44^−/−^ ST/WT mice was 21 μm ± 0.3 μm and 22 μm ± 0.7 μm, respectively. The mean cone IS and OS length was not significantly different between the two groups, suggesting that CD44 does not affect cone outer segment development. At 4 weeks of age, cone IS and OS length of ST/WT retinas increased to 27 μm ± 0.8 μm. In contrast, in CD44^−/−^ ST/WT mice, the cone IS and OS length was 19 μm ± 0.9 μm (ANOVA, *P* ≤ .001; vs. age-matched ST/WT control). There was a significant increase to 25 μm ± 1 μm in cone IS and OS length at 8 weeks (ANOVA, *P* ≤ .001; week 4 vs. week 8 in CD44^−/−^ ST/WT mice), even if the ST/WT cone IS and OS length was not reached. At 8 and 12 weeks of age, the cone IS and OS lengths were significantly reduced in CD44^−/−^ ST/WT mice compared to ST/WT (ANOVA, *P* ≤ .05). In ST/ST and CD44^−/−^ ST/ST mice, the cone IS and OS length progressively degenerated over time (Fig. 3B, right panel).

Photoreceptor degeneration is accompanied by changes in downstream neurons [41]. To evaluate the effect of CD44 loss on rod bipolar, cone bipolar, and horizontal neurons, we visualized these cells by immunostaining for protein kinase c alpha (PKC-α), secretagogin (SCGN), and calbindin, respectively. In CD44^−/−^ ST/WT retinas, rod bipolar cell dendrites were shorter and less bushy (vs. age-matched ST/WT control). In ST/ST and CD44^−/−^ST/ST retinas, the retraction of rod bipolar cell dendrites was further progressed (Fig. 3C). We next quantified the observed changes in rod bipolar cell dendrites. In CD44^−/−^ST/WT mice, the dendrite area was significantly reduced compared with ST/WT mice (ANOVA, * *P* ≤ .05). In ST/ST and CD44^−/−^ST/ST mice, the dendritic area was also significantly reduced compared to age-matched ST/WT and CD44^−/−^ ST/WT mice, respectively (ANOVA, * *P* ≤ .05). There was no significant difference between CD44^−/−^ ST/ST and ST/ST mice. (Fig. 3D). Cone bipolar cell dendrites were similar between CD44^−/−^ ST/WT and ST/WT retinas. In ST/ST and CD44^−/−^ ST/ST retinas, the cone bipolar cell dendrites were modestly retracted (Fig. 3E). We next quantified the observed changes in cone bipolar cell dendrites. In ST/ST and CD44^−/−^ ST/ST mice, the dendritic area was significantly reduced compared to age-matched ST/WT and CD44^−/−^ ST/WT mice, respectively (ANOVA, * *P* ≤ .05). There were no significant differences between CD44^−/−^ ST/WT and ST/WT retinas as well as between CD44^−/−^ ST/ST and ST/ST retinas (Fig. 3F). In CD44^−/−^  ST/WT and ST/WT retinas, horizontal cell processes were bushy. In ST/ST and CD44^−/−^ ST/ST retinas, the horizontal cell processes were retracted (Fig. 3G). We next quantified the observed changes in horizontal cell processes. In ST/ST and CD44^−/−^ ST/ST mice, the area was significantly reduced compared to age-matched ST/WT and CD44^−/−^ ST/WT mice, respectively (ANOVA, *** *P* ≤ .001). There were no significant differences between CD44^−/−^ ST/WT and CD44^−/−^ ST/ST retinas compared to ST/WT and ST/ST retinas, respectively (Fig. 3H). In conclusion, loss of CD44 results in a slight decrease in ONL thickness, cone IS and OS length, and dendritic retraction of rod bipolar neurons.

### Loss of CD44 impairs scotopic and mesopic retinal function

**Fig. 4:**
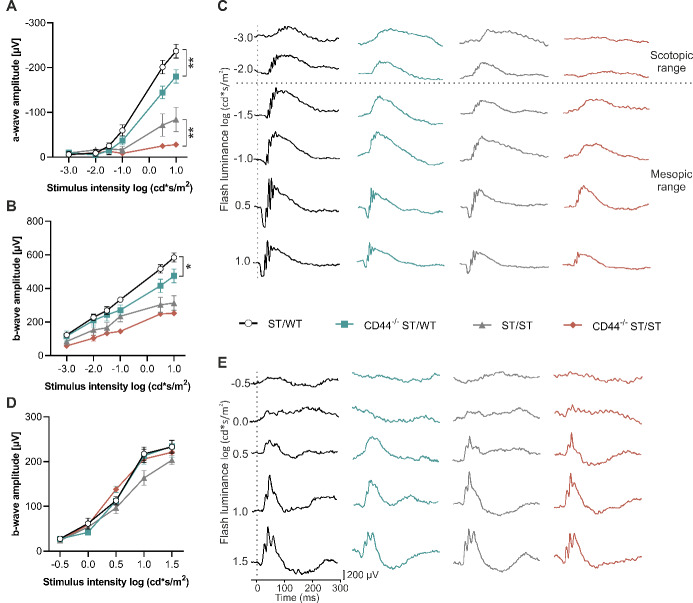
Decreased scotopic and mesopic retinal function in CD44-deficient mice. (**A-E**) ERGs were recorded from 8-week-old ST/WT, CD44^−/−^ ST/WT, ST/ST, and CD44^−/−^ ST/ST mice. (**A**) Scotopic and mesopic a-wave amplitudes. A-wave amplitudes of CD44^−/−^ ST/WT and CD44^−/−^ ST/ST at 0.5 and 1.0 log (cd*s/m^2^) were significantly decreased compared to ST/WT and ST/ST, respectively. (**B**) Scotopic and mesopic b-wave amplitudes. At the highest measured light intensity of 1.0 log (cd*s/m^2^), the b-wave amplitude of CD44^−/−^ ST/WT mice was significantly decreased compared to ST/WT mice. (**C**) Representative ERG responses in the scotopic and mesopic range. (**D**) Photopic b-wave amplitudes. B-wave amplitudes were similar between all groups. (**A**, **B**,** D**) Data, presented as mean ± SEM, were compared by ANOVA, * *P* < .05; ** *P* < .01. *N* = 7 per group. (**E**) Representative ERG responses in the photopic range

To test whether CD44 impacts retinal function, full-field single-flash electroretinography (ERG) responses were recorded in 8-week-old mice. In CD44^−/−^ ST/WT mice (vs. age-matched ST/WT control), the a-wave (negative deflection), generated by photoreceptor cells, was significantly decreased at light intensities of 0.5 and 1.0 log (cd*s/m^2^) (*P* ≤ .001). In both CD44^−/−^ ST/ST and ST/ST mice (vs. age-matched CD44^−/−^ ST/WT and ST/WT, respectively), the a-wave was reduced (Fig. 4A, C). The a-wave of CD44^−/−^ ST/ST mice was significantly smaller compared to ST/ST mice at light intensities of 0.5 and 1.0 log (cd*s/m^2^) (Fig. 4A). In CD44^−/−^ ST/WT mice, the b-wave amplitude (positive deflection), generated by bipolar cells, was significantly decreased at a light intensity of 1.0 log (cd*s/m^2^) compared to ST/WT mice (ANOVA, *P* = .03). The b-wave amplitudes of CD44^−/−^ ST/ST mice were not significantly decreased at all light intensities compared to ST/ST mice (Fig. 4B). After light-adaption, to derive cone-response, the b-wave amplitude was measured. There was no significant difference between the groups. ERG recordings of 8-week-old ST/WT, CD44^−/−^ ST/WT, ST/ST, and CD44^−/−^ ST/ST mice showed an increase in the b-wave amplitude with each increased intensity (Fig. 4D, E). In summary, the absence of CD44 leads to a decreased scotopic and mesopic ERG response.

### Loss of CD44 results in downregulation of the SLC1A2 glutamate transporter in Müller cells

**Fig. 5:**
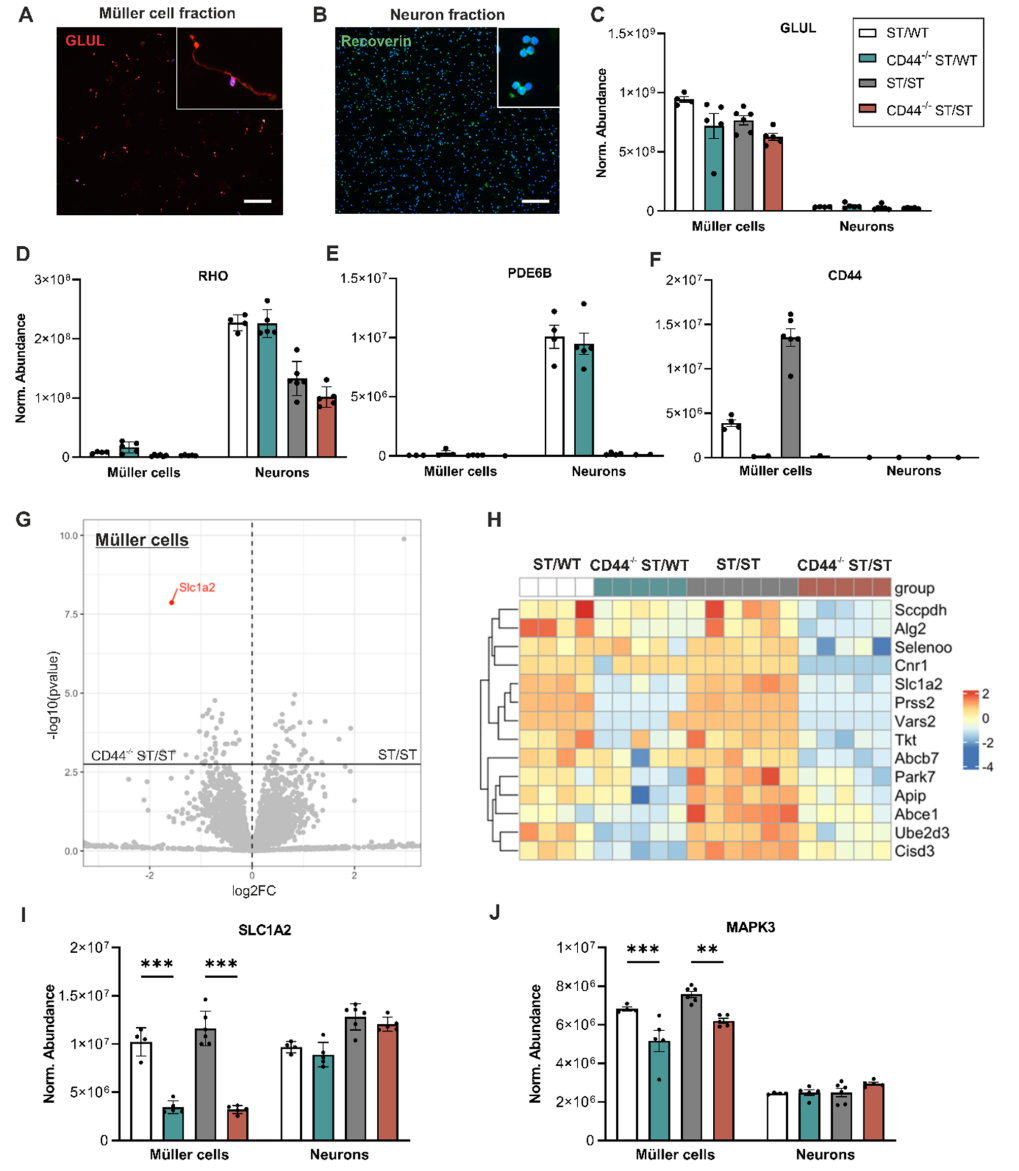
Proteomic profiling of Müller cells and neurons. MACS enriched Müller cells and neuron cell fractions were subjected to label-free liquid chromatography-tandem mass spectrometry (LC-MS/MS)-based proteomics. ST/WT *N* = 4, CD44^−/−^ ST/WT, CD44^−/−^ ST/ST *N* = 5, ST/ST *N* = 6. (**A**) Representative image of Müller cell fraction immunostained for glutamine synthetase (GLUL) and counterstained with Hoechst 33,342. (**B**) Representative image of neuron fraction immunostained for recoverin and counterstained with Hoechst 33,342. Scale bar, 50 μm. **(C-F)** Quantitative analysis of GLUL (Müller cell marker) (**C**), rhodopsin (RHO) (rod photoreceptor marker) (**D**), PDE6B (**E**) and CD44 (**F**). (**G**) Volcano plot showing differentially expressed proteins between ST/ST and CD44^−/−^ ST/ST Müller cells. Red dot, solute carrier family 1 member 2 (SLC1A2) with fold change > 5, and FDR < 0.1. (**H**) Heat map representation of Müller cell-specific proteins involved in metabolic processes. (**I**,** J**) Quantitative analysis of SLC1A2 (**I**) and MAPK3 (**J**). Data, presented as mean ± SEM, were compared by ANOVA. * *P* ≤ .05; ** *P* ≤ .01; *** *P ≤* .001

Since Müller cells make up only a small fraction of cells in the murine retina [42], proteomic studies of whole retinas cannot provide Müller cell-specific information. Therefore, to identify proteins dysregulated in Müller cells, we performed proteomic analysis on Müller cells and neurons isolated from 8-week-old ST/WT, ST/ST, CD44^−/−^ ST/WT, and CD44^−/−^ST/ST retinas using a multistep magnetic-activated cell sorting (MACS) procedure. To assess the purity and morphology, freshly isolated Müller cells and neurons were immunostained for GLUL and Recoverin, respectively. Almost all of the cells were double positive for Hoechst and the cell type-specific antibody GLUL or Recoverin (Fig. 5A, B), demonstrating the high purity of the respective fractions. Importantly, isolated Müller cells retained their characteristic polar morphology (Fig. 5A, **insert**).

The label-free quantitative LC-MS/MS-based proteomic analysis revealed a total of approximately 5200 different proteins in both the Müller cell and neuronal fraction. To assess the quality of both the proteomic data and the MACS, we quantitatively compared the abundance of GLUL, Rhodopsin (RHO), CD44, and PDE6B across the 4 groups and the two cell fractions (Fig. 5C-F). GLUL, a Müller cell marker, was highly expressed in Müller cells of all groups and reduced in neurons (Fig. 5C). Rhodopsin (RHO), a rod photoreceptor marker, was highly expressed in neurons and reduced in Müller cells (Fig. 5D). RHO expression was highest in neurons from ST/WT and CD44^−/−^ ST/WT mice and slightly reduced in ST/ST and CD44^−/−^ ST/ST mice because of the diminished number of rods. PDE6B was highly expressed in neurons from ST/WT and CD44^−/−^ ST/WT mice and absent in neurons from ST/ST and CD44^−/−^ ST/ST mice, confirming the loss of PDE6B. PDE6B was not detected in Müller cells (Fig. 5E). CD44 was expressed in Müller cells from ST/WT, increased in Müller cells from ST/ST mice and absent in Müller cells from CD44^−/−^ST/WT and CD44^−/−^ ST/ST mice, confirming on the one hand the upregulation of CD44 in ST/ST retinas and on the other hand the loss of CD44 in CD44^−/−^ ST/WT and CD44^−/−^ ST/ST retinas. CD44 was not detected in neurons (Fig. 5F).

A volcano plot was used to visualize proteins with statistical differences in expression (adj. *P*-value < 0.5) between ST/ST and CD44^−/−^ ST/ST Müller cells. Solute carrier family 1 member 2 (SLC1A2), also known as excitatory amino acid transporter 2 (EAAT2) or glutamate transporter 1 (GLT-1) [43], was significantly downregulated in Müller cells from CD44^−/−^ ST/ST mice (vs. ST/ST) (Fig. 5G). Since SLC1A2 is essential for glutamate homeostasis and metabolism, we visualized the expression of Müller cell-specific genes involved in metabolic processes as identified by the gene ontology (GO) term 0008152 (Fig. 5H). Importantly, proteins related to metabolic processes were highly expressed in ST/WT and ST/ST (Fig. 5H). SLC1A2 is significantly downregulated in the Müller cell fractions of CD44^−/−^ ST/WT and CD44^−/−^ ST/ST retinas compared to ST/WT and ST/ST retinas, respectively (ANOVA, *P* ≤ .001), whereas it was not altered in the neuron fraction (Fig. 5I). Since CD44 signaling leads to activation of the MAPK pathway [44], we analyzed MAPK3 expression in Müller cells and neurons. Interestingly, MAPK3 was significantly downregulated in Müller cells of CD44^−/−^ ST/WT and CD44^−/−^ ST/ST retinas compared to ST/WT and ST/ST retinas, respectively (ANOVA, *P ≤* .001 and *P* = .001, respectively). Furthermore, we found that MAPK3 was slightly but not significantly upregulated in ST/ST compared to ST/WT neurons (ANOVA, *P* = .3) (Fig. 5J). These results may indicate that CD44 regulates SLC1A2 via the MAPK3 pathway.

**Fig. 6:**
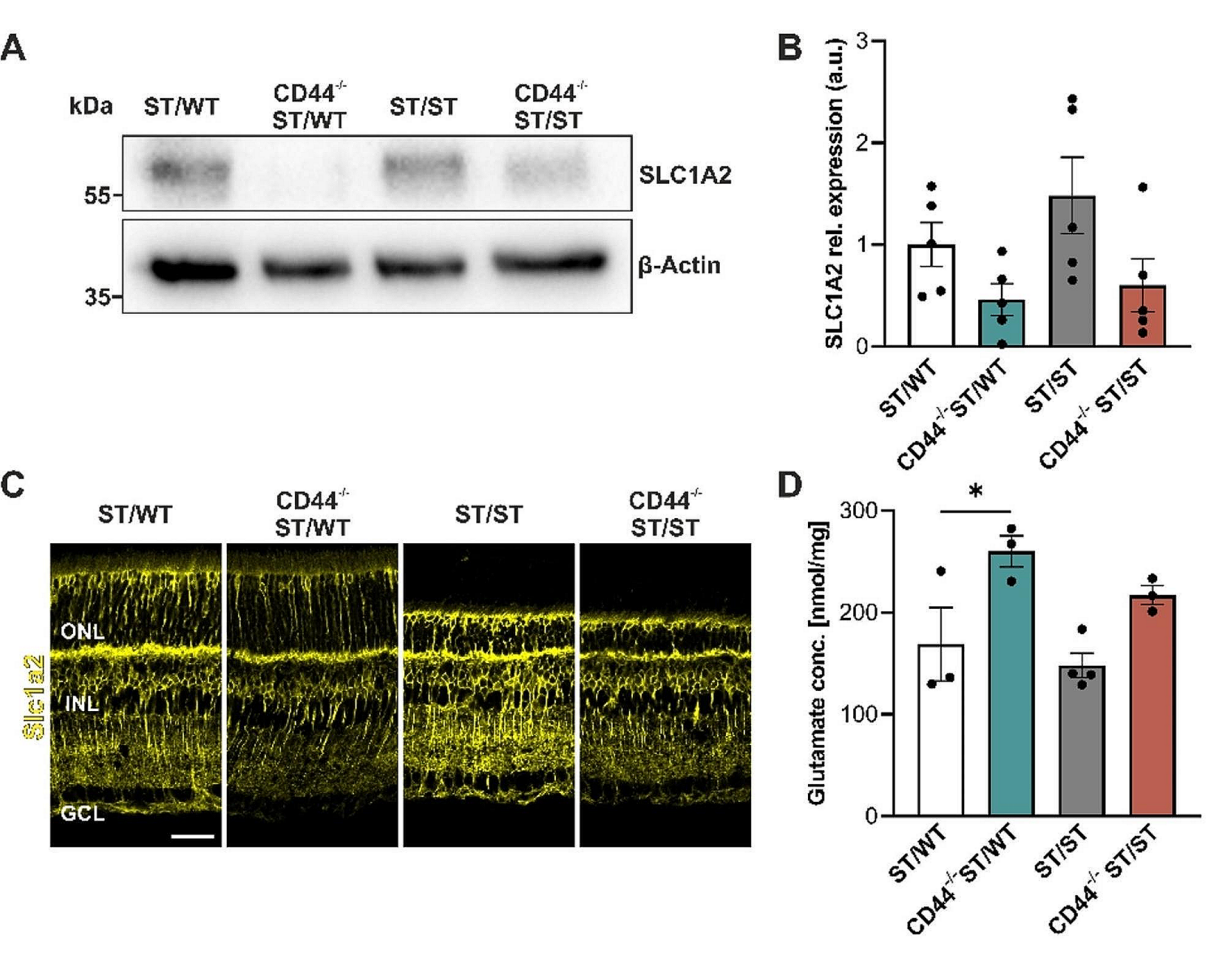
Validation of decreased SLC1A2 expression in CD44-deficient mice. (**A-D**) Retinas from ST/WT, CD44^−/−^ ST/WT, ST/ST, and CD44^−/−^ ST/ST were analyzed at 8 weeks of age. (**A**) Representative SLC1A2 immunoblot of retinal lysates. β-Actin was used as a loading control. (**B**) Quantitative analysis of SLC1A2 immunoblots. Data are presented as mean ± SEM. *N* = 5 per group. (**C**) Representative images of retinal sections immunostained for the glutamate transporter SLC1A2. SLC1A2 is expressed in Müller cells and cones. Scale bar, 35 μm. (**D**) Fluorometric glutamate assay revealed a significantly higher glutamate concentration in CD44^−/−^ ST/WT retinas compared to ST/WT. Data, presented as mean ± SEM, were compared by ANOVA, * *P* ≤ .05; ST/WT, CD44^−/−^ ST/WT, CD44^−/−^ ST/ST *N* = 3, ST/ST *N* = 4. ONL, outer nuclear layer; INL, inner nuclear layer; GCL, ganglion cell layer

Next, we validated the decreased expression of SLC1A2 found by proteome analysis of Müller cells using immunoblots, which confirmed SLC1A2 downregulation in CD44^−/−^ ST/WT and CD44^−/−^ ST/ST retinas (Fig. 6A). The quantitative analysis indicates a trend consistent with the immunoblot results; however, the differences observed were not statistically significant (Fig. 6B). Additionally, SLC1A2 also appeared to be downregulated in the immunohistochemistry analysis (Fig. 6C). Co-immunostaining for GLUL (Müller cells) and SLC1A2 in retinal sections from 12-week-old CD44^−/−^ ST/WT mice showed that SLC1A2 is expressed in Müller cells (Fig. S4A). Based on the expression pattern and previous studies [45, 46], SLC1A2 seems to be also expressed in cones, bipolar, and amacrine cells. To determine whether the observed downregulation of the glutamate transporter SLC1A2 impacts retinal glutamate levels, a fluorometric glutamate assay was utilized. The highest glutamate level of 260 nmol/mg was detected in CD44^−/−^ ST/WT retinas compared to 169 nmol/mg in ST/WT retinas (ANOVA, *P* < .05). Glutamate concentration in CD44^−/−^ ST/ST retinas was also increased to 217 nmol/mg compared to 148 nmol/mg in ST/ST retinas, but this difference was not significant (ANOVA, *P* = .1) (Fig. 6D).

### Increased pro-inflammatory response in *Cd44*^*−/−*^*Pde6b*^*STOP/STOP*^mice

**Fig. 7:**
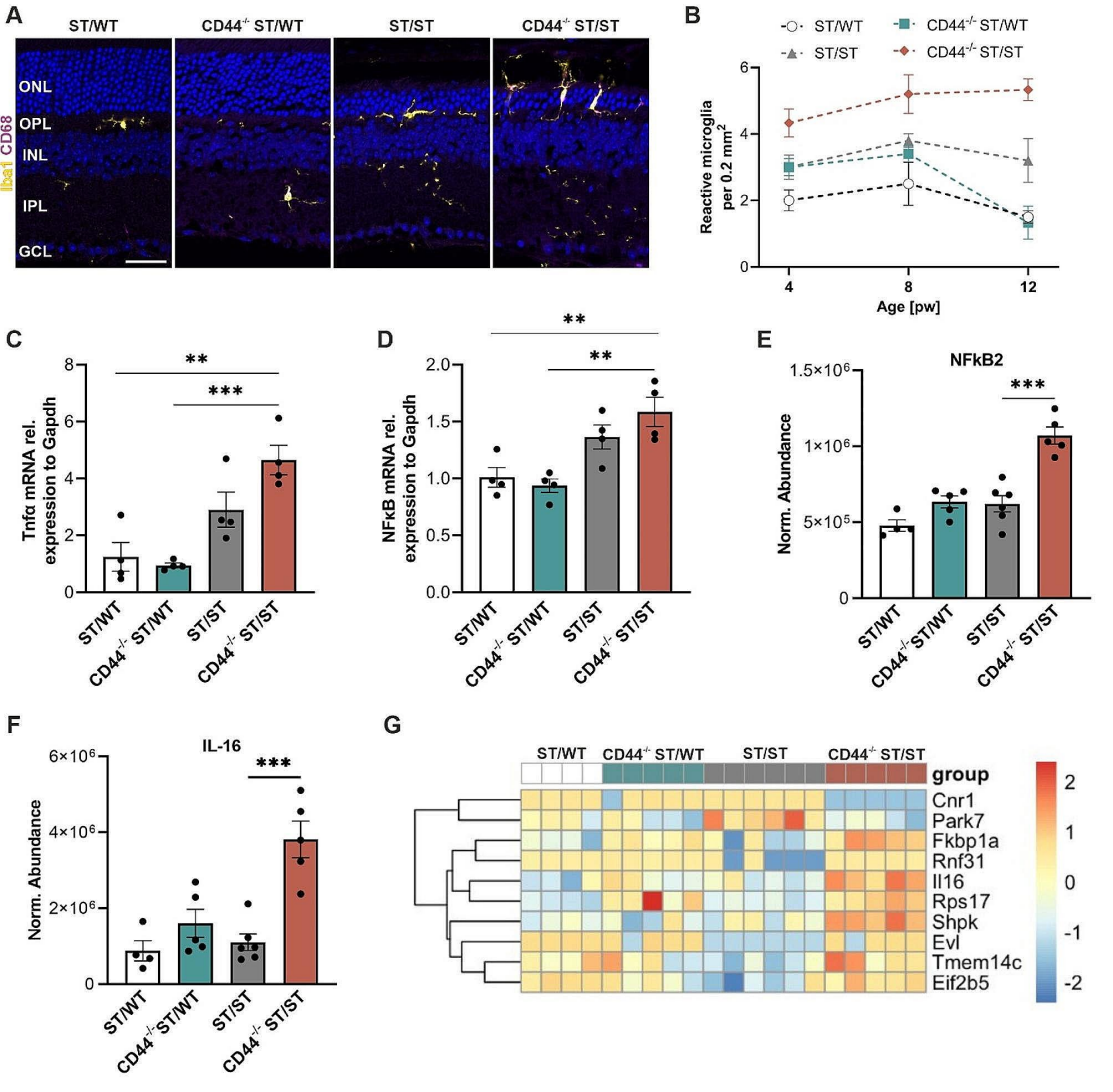
Increased neuroinflammation in CD44^−/−^ST/ST mice. (**A**) Representative images of retinal sections from 12-week-old mice immunostained for Iba1 and CD68 to visualize reactive microglia/ macrophages. Scale bar, 35 μm. (**B**) Quantification of reactive microglia at 4, 8, and 12 weeks of age. pw 4, *N* = 5 for ST/WT, *N* = 6 for CD44^−/−^ ST/WT, ST/ST, and CD44^−/−^ ST/ST. pw 8, *N* = 4 for ST/WT, *N* = 5 for CD44^−/−^ ST/WT, ST/ST, and CD44^−/−^ ST/ST. pw 12, *N* = 8 for ST/WT, *N* = 6 for CD44^−/−^ ST/WT, and CD44^−/−^ ST/ST, *N* = 5 for ST/ST. (**C-D**) Quantitative mRNA analysis of TNFα (**C**), and NFκB (**D**) by qRT-PCR at 8 weeks of age. *N* = 4 per group. (**E-F**) Quantitative analysis of NFκB2 (**E**) and IL-16 (**F**) in isolated Müller cells. ST/WT *N* = 4, CD44^−/−^ ST/WT, CD44^−/−^ ST/ST *N* = 5, ST/ST *N* = 6. (**B-F**) Data, presented as mean ± SEM, were compared by ANOVA. ** *P* ≤ .01; *** *P* < .001. (**G**) Heat map representation of Müller cell-specific proteins involved in immune system processes. ONL, outer nuclear layer; OPL, outer plexiform layer; INL, inner nuclear layer; IPL, inner plexiform layer; GCL, ganglion cell layer

Inflammation, a common feature of retinal degeneration [47], is characterized by reactive microglia, and an upregulation of several pro-inflammatory molecules [48]. To evaluate whether loss of CD44 leads to changes in the pro-inflammatory response, we quantified the total number of reactive microglia in retinal sections of 4-, 8-, and 12-week-old-mice immunostained for IBA1 (microglia/macrophages) [49, 50] and CD68 (reactive microglia/macrophages) [51]. At 12 weeks of age, microglia had a ramified morphology and were located between the IPL and the OPL of ST/WT and CD44^−/−^ ST/WT mice. In contrast, in retinas from 12-week-old CD44^−/−^ ST/ST and ST/ST mice, microglia were reactive with extending processes and were located in the ONL (Fig. 7A). For quantification, microglia positive for Hoechst 33,342, Iba1, and CD68 were counted. CD44^−/−^ ST/ST retinas showed higher numbers of reactive microglia than ST/ST retinas at 4, 8, and 12 weeks of age (ANOVA, *P* = .056, *P* = .043, and *P* = .027, respectively). In CD44^−/−^ ST/ST mice, the number of microglia increased in parallel with disease progression (Fig. 7B).

Reactive phagocytic microglia release pro-inflammatory factors such as TNFα, which activate NF-κB signaling, leading to a cascade of downstream effects [52, 53]. The mRNA level of Tnfα was significantly elevated in 8-week-old CD44^−/−^ ST/ST retinas compared to ST/WT and CD44^−/−^ ST/WT retinas (Fig. 7C). Additionally, Tnfα was also increased in CD44^−/−^ ST/ST retina compared to ST/ST, although this difference was not significant (Fig. 7C). The mRNA level of NF-κB was significantly higher in 8-week-old CD44^−/−^ ST/ST retinas compared to ST/WT and CD44^−/−^ ST/WT retinas. There were no significant differences between CD44^−/−^ ST/ST and ST/ST retinas (Fig. 7D). In conclusion, increased activation of microglia in CD44^−/−^ ST/ST mice was associated with increased expression of the proinflammatory factors TNFα and NF-kB.

To understand the inflammatory response of Müller cells [54], we analyzed NFkB2 and pro-interleukin-16 (IL-16) in our MAC-sorted proteome data. We found that pro-inflammatory NFkB2 and IL-16 were significantly increased in Müller cells of CD44^−/−^ ST/ST retinas compared to ST/ST (Fig. 7E, F). We also visualized the expression of genes involved in immune system processes (GO term: 0002376) (Fig. 7G). There were no significant changes between ST/WT and CD44^/−^ ST/WT retinas. Most of the proteins were highly upregulated in Müller cells of CD44^−/−^ ST/ST retinas (Fig. 7G). These data show that CD44 reduces the retinal pro-inflammatory response to photoreceptor degeneration.

## Discussion

Our data demonstrate that CD44 is dramatically upregulated in various RP mouse models. CD44 is the primary receptor for hyaluronic acid (HA) [55], which is a critical component of the extracellular matrix (ECM) [55, 56]. HA often accumulates in neurodegenerative diseases (e.g., multiple sclerosis, Alzheimer’s diseases) [57]. The HA concentration also appeared to be increased in our RP mouse model, indicating a positive feedback loop and a restructuring of the ECM composition in response to photoreceptor degeneration. Changes in the ECM composition might be important to meet the requirements of adjacent retinal cells and to enhance the connectivity between the ECM and the cytoskeleton of Müller cells [6]. Alterations in the interphotoreceptor matrix, the ECM between the outer limiting membrane and the RPE, might also impair the exchange of metabolites between Müller cells, photoreceptors, and RPE [5, 23]. The greater HA accumulation in *Cd44*-deficient retinas might be a compensatory mechanism for the loss of CD44; in inflamed joint tissue from CD44-deficient mice following collagen-induced arthritis, HA concentration was also increased [58].

Regarding the downstream effects of CD44, our findings reveal a previously unknown role for CD44 in the retinal glutamate homeostasis. In healthy retinas, Müller cells contribute to the removal of extracellular glutamate, thereby protecting neurons from excitotoxicity [59–61]. The uptake and metabolization of glutamate are also part of the glutamate-glutamine cycle and is linked to the metabolic support of photoreceptors [2, 62]. In *Cd44*-deficient mice, we observed a significant downregulation of the glutamate transporter SLC1A2 in Müller cells and increased retinal glutamate levels. We also observed some photoreceptor loss in these mice, which might be due to glutamate-driven neurotoxic effects and/or inadequate matching of metabolic needs [59–61]. Although the performed glutamate assay does not distinguish between intracellular and extracellular glutamate, we assume that downregulation of SLC1A2 results in impaired glutamate uptake and thus, significant accumulation of extracellular glutamate. Moreover, Müller cells rapidly convert glutamate to glutamine and α-ketoglutarate after uptake, minimizing their intracellular glutamate concentration [62]. It has been suggested that SLC1A2 is not expressed in Müller cells but in cones, bipolar, and amacrine cells [45, 46, 63] and that glutamate aspartate transporter 1 (GLAST1) is the most abundant and significant glutamate transporter in Müller cells [45, 59, 64]. However, our MAC-sorted proteomics and immunohistochemistry data demonstrate that SLC1A2 is indeed expressed in Müller cells and impacts glutamate homeostasis.

CD44 has previously been linked to inflammation [65–67]. For example, CD44 can facilitate the recruitment of neutrophils and macrophages to sites of inflammation [68, 69]. Administration of anti-CD44 antibodies inhibited inflammation in mouse models of inflammatory bowel disease, collagen- and proteoglycan-induced arthritis, cutaneous inflammation, and IL-2-induced vascular leak [70]. In addition, CD44 deficiency in a Parkinson’s disease mouse model decreased activation of microglia and astrocytes [71]. This has led to the suggestion that CD44 should be considered as a target in inflammatory diseases. However, results of studies using *Cd44*-deficient mice showed increased lung inflammation in response to *E. coli*-induced pneumonia [72]. In line with this finding, we show that loss of CD44 in RP retinas leads to an increased immune response, characterized by activation of microglia and Müller cells, the initiation of the inflammatory NFκB pathway, and the release of proinflammatory cytokines (e.g., IL-16). The significantly upregulated proinflammatory factor NF-κB in CD44^*−/−*^ ST/ST retinas may also further impede Müller cell glutamate uptake [73, 74]. It has also been suggested that increased numbers of reactive microglia may increase synaptic glutamate levels [75, 76] while downregulating the expression and functionality of glutamate transporters such as SLC1A2 [75].

Since we used a global CD44 knockout mouse model rather than a Müller cell-specific knockout, it is important to note that CD44, while predominantly expressed in Müller cells, may also have functional implications in other retinal cell types. Thus, while the observed phenotype likely involves alterations in Müller cell function due to CD44 loss, contributions from additional retinal cells cannot be discounted. Further investigations, perhaps utilizing cell-specific knockout models, are necessary to delineate the precise roles of CD44 in different retinal cell types and their collective impact on the observed phenotype.

## Materials and methods

### Animals

All animal experiments were performed in accordance with the ARVO statement on the use of animals in ophthalmic and vision research and were approved by the local authorities (Regierung von Oberbayern, ROB-55.2-2532.Vet_02-18-143). Mice were kept under standard conditions on a 12 h light/ dark cycle with access to water and food *ad libitum*. *Pde6b*^*STOP*^ mice were generated in the Barbara & Donald Jonas Stem Cells Laboratory, Columbia University, USA [77]. B6.129(Cg)-*Cd44*^*tm1Hbg*^/J mice (referred to as CD44^−/−^) were purchased from the Jackson Laboratory (#005085). These mice carry a neomycin resistance/lacZ cassette that disrupts exon 1 and part of intron 1, resulting in a complete loss of CD44 transcription [14]. *Cd44*^*−/−*^ mice were crossed with *Pde6b*^*STOP*^ mice to generate *Cd44*^*−/−*^*Pde6b*^*STOP/STOP*^ (referred to as CD44^−/−^ ST/ST) and *Cd44*^*−/−*^*Pde6b*^*STOP/+*^ (referred to as CD44^−/−^ ST/WT) mice.

### Immunohistochemistry

Retinal sections were incubated in primary antibodies (Table 1) in blocking solution (5% chemiblocker #2170, MerckMillipore; and 0.3% Triton^®^ X-100 diluted in PBS) overnight at 4 °C. Subsequently, sections were incubated in secondary antibodies (Table 1) in PBS containing 3% chemiblocker for 1.5 h at room temperature. For nuclear counterstaining, sections were incubated for 5 min in 5 µg/ml Hoechst 33,342 (#H1399, Invitrogen).

**Table 1:** Primary and secondary antibodies

Antibody		Host species	Dilution (IHC)	Dilution (WB)	Supplier	Catalog number
Β-Actin-Peroxidase	Mouse		-	1:6000	Sigma-Aldrich	A3854-200UL
Calbindin D-28k	Mouse		1:800	-	Swant	300
CD44	Rat		1:400	-	BD Pharmingen	550,538
CD44	Rabbit		-	1:2000	Abcam	ab28364
CD68	Rat		1:500	-	Bio-Rad	MCA1957T
Cone Arrestin (ARR3)	Rabbit		1:1000	-	Merck	AB15282
GFAP	Mouse		1:1000	1:800	Sigma-Aldrich	G3893
GLUL	Rabbit		1:2000	1:2000	Abcam	ab228590
Iba1	Rabbit		1:1000	-	VWR / Fujifilm Wako	WAKO019-19741
Pde6b	Mouse		-	1:400	Santa Cruz	sc77486
PKCα	Mouse		1:1000	-	Santa Cruz	sc-8393
Recoverin	Rabbit		1:1000	-	Sigma-Aldrich	AB5585
Secretagogin	Rabbit		1:5000	-	Prof. Dr. Wagner (University of Vienna)	
Slc1a2	Rabbit		1:50	1:1000	Cell Signaling	20,848
488-Goat anti-Rat	Goat		1:1000	-	Thermo Fisher	A-11,006
488-Goat anti-Rabbit	Goat		1:1000	-	Thermo Fisher	A-11,070
555-Goat anti-Mouse	Goat		1:1000	-	Thermo Fisher	A-21,425
555-Goat anti-Rat	Goat		1:1000	-	Jackson	112-165-143
647-Goat anti-Rabbit	Goat		1:1000	-	Thermo Fisher	A-21,245
anti-Rabbit HRP	Mouse		-	1:2000	Santa Cruz	sc-2357
anti-Mouse HRP	Mouse		-	1:2000	Santa Cruz	sc-516,102

### Hematoxylin and eosin staining

Retinal sections were sequentially incubated for 5 min in 100% ethanol, followed by 5 min in 96% ethanol, and then 5 min in 70% ethanol. Subsequently, sections were briefly immersed in desalted water for 3 s before being incubated for 5 min in hematoxylin solution (Sigma Aldrich #MHS32-1 L). Slides were then washed for 5 min in desalted water. Following this, slides were incubated for 5 min in eosin Y solution (Sigma Aldrich #HT110216-500ML). Afterward, slides were dipped several times in desalted water and subsequently sequentially immersed for 3 s each in 70% ethanol, 96% ethanol, and finally 100% ethanol. Lastly, slides were dipped in xylene several times and mounted directly using Eukitt quick-hardening mounting medium (Sigma Aldrich #03989 − 100).

### Imaging and quantification

For quantitative analyses, the retinal images were acquired with the compact fluorescence microscope KEYENCE BZ-X800. Outer nuclear layer (ONL) thickness was measured at 300 μm from the optic nerve on the ventral side using the Fiji software. For the quantification of cone inner (IS) and outer segment (OS) length, the lengths of three cone IS and OS were measured at a distance of around 300 μm from the optic nerve. Rod bipolar cell, horizontal cell, and cone bipolar cell dendrite areas close to the optic nerve were quantified as pixels in the outer plexiform layer using Fiji. To visualize the ONL thickness, spider plots were generated. Retinal images were acquired from both the ventral and dorsal sides, and ONL thickness was measured at 250 μm, 500 μm, 750 μm, and 1000 μm from the optic nerve on both sides. For the quantification of reactive microglia, cells that were positive for Hoechst, IBA1, and CD68 were counted in areas close to the optic nerve. All data were plotted using GraphPad Prism 9.3. Data are expressed as mean ± standard error of mean using an ANOVA analysis. *P* < .05 was considered statistically significant (*P < .05; **P < .01; ***P < .001). The N values refer to the number of individual animals for the respective genotype and are listed in the respective figure legends.

### Immunoblot

Retinas were homogenized using M-PER Mammalian Protein Extraction Reagent (Thermo #78,503) containing protease inhibitor (Sigma #11,697,498,001) and Phosphatase Inhibitor Cocktail (Cell Signaling; #5870) with a Branson Sonifier W-450D at 40% amplitude. Proteins (20 µg per sample) were separated by SDS-PAGE and transferred to a 0.45 μm polyvinylidene difluoride (PVDF) membrane for 90 min at 90 V. Membranes were blocked in 5% non-fat dry milk in Tris-buffered saline with Tween^®^20 (TBS-T) for 1 h at RT. Primary antibodies (Table 1) were incubated in 5% non-fat dry milk overnight at 4 °C. Membranes were washed and incubated with corresponding HRP secondary antibody (Table 1) for 1 h at RT. Proteins were detected using Immobilon Forte Western HRP substrate (Millipore #WBLUF0100) and imaged using a Bio-Rad ChemiDoc MP imager.

### RT-qPCR

RNA was isolated from retinas with the NucleoSpin^®^ RNA kit (Macherey-Nagel) according to the manufacturer’s protocol. 0.5–1 µg of total RNA was used for cDNA synthesis using the RevertAid First Strand cDNA Synthesis Kit (Therme Fisher Scientific) following the manufacturer’s instructions. The obtained cDNA was diluted 1:5 with distilled water and used for quantitative real-time PCR using the PowerUp™ SYBR™ Green Master Mix (Thermo Fisher Scientific) on a QuantStudio™ 5 Real-Time PCR System (Thermo Fisher Scientific). Exon-spanning primer pairs were designed to hybridize exclusively to the desired transcript and to avoid genomic contamination using the UCSC genome browser. Primer specificity was verified by melting curve analysis and gel electrophoresis of qPCR products. The following primers were used: *Cd44* 5’-ACGAGGAGGAGGTGTGATGT- 3’ (Forward), 5’-GTGGCTTTTTGAGGGGTTC-3’ (Reverse); *Gapdh* 5’-CATCACTGCCACCCAGAAGACTG-3‘ (Forward), 5’-ATGCCAGTGAGCTTCCCGTTCAG-3‘ (Reverse); *Gfap* 5’-CACCTACAGGAAATTGCTGGAGG-3‘ (Forward), 5’-CCACGATGTTCCTCTTGAGGTG-3‘ (Reverse); *NF-κB* 5’-GCTGCCAAAGAAGGACACGACA-3’ (Forward), 5’-GGCAGGCTATTGCTCATCACAG-3’ (Reverse); *Tnf-α* 5’-TCTTCTCATTCCTGCTTGTGG-3’ (Forward), 5’-GGTCTGGGCCATAGAACTGA-3’ (Reverse).

### ERG

ERG analysis was performed at 8 weeks of age according to previously described procedures [29].

### Hyaluronan ELISA

Retinas were homogenized in 95 µl M-PER Mammalian Protein Extraction Reagent (Thermo #78,503), 4 µl protease inhibitor (Sigma #11,697,498,001), and 1 µl Phosphatase Inhibitor Cocktail (Cell Signaling; #5870) with a Branson Sonifier W-450D. The lysates were diluted 1:4 with distilled water and 50 µl of the sample lysates were used per well. The Quantikine™ Hyaluronan ELISA assay (R&D Systems) was conducted according to the manufacturer’s protocol.

### Statistics

All data were plotted using GraphPad Prism 9.3. ANOVA was used to analyze data. *P* < .05 was considered statistically significant (*P < .05; **P < .01; ***P < .001). The N values refer to the number of individual animals for the respective genotype.

### Proteomic profiling of MACS enriched retinal cell types

MACS was performed according to previously described procedures [78]. Isolated retinal cell populations were analyzed with label-free quantitative liquid chromatography coupled-mass spectrometry (LC-MSMS), followed by processing of raw data with Proteome Discoverer 2.4 SP1 software as described [78, 79]. Quantification of proteins, after precursor recalibration, was based on intensity values (at RT apex) for the top three unique peptides per protein. Peptide abundance values were normalized on the total peptide amount. The protein abundances were calculated averaging the abundance values for admissible peptides. The final protein ratio was calculated using median abundance values of three to five biological replicates in a non-nested design.

### Glutamate assay

After protein extraction from retinal lysates and quantification (as described in Immunoblot), 2 µl of the lysate was diluted with 18 µl distilled water. Subsequently, 1.5 µl of the diluted samples were added into wells of a 96-well plate. The glutamate concentration per retina was measured using the PicoProbe™ Glutamate Assay Kit (Fluorometric) (BioVision) according to the manufacturer’s protocol.

### Electronic supplementary material

Below is the link to the electronic supplementary material.

Supplementary Material 1

Supplementary Material 2

## Data Availability

No datasets were generated or analysed during the current study.

## References

[1] Tomita Y, Qiu C, Bull E, Allen W, Kotoda Y, Talukdar S, et al. Müller glial responses compensate for degenerating photoreceptors in retinitis pigmentosa. Exp Mol Med. 2021;53(11):1748–58. 34799683 10.1038/s12276-021-00693-w PMC8639781

[2] Bringmann A, Grosche A, Pannicke T, Reichenbach A. GABA and glutamate uptake and metabolism in retinal glial (Müller) cells. Front Endocrinol (Lausanne). 2013;4:48. 23616782 10.3389/fendo.2013.00048 PMC3627989

[3] Toft-Kehler AK, Skytt DM, Kolko M. A perspective on the Müller Cell-Neuron Metabolic Partnership in the Inner Retina. Mol Neurobiol. 2018;55(6):5353–61. 28929338 10.1007/s12035-017-0760-7

[4] Viegas FO, Neuhauss SCF. A metabolic Landscape for maintaining retina Integrity and function. Front Mol Neurosci. 2021;14:656000. 33935647 10.3389/fnmol.2021.656000 PMC8081888

[5] Ishikawa M, Sawada Y, Yoshitomi T. Structure and function of the interphotoreceptor matrix surrounding retinal photoreceptor cells. Exp Eye Res. 2015;133:3–18. 25819450 10.1016/j.exer.2015.02.017

[6] Al-Ubaidi MR, Naash MI, Conley SM. A perspective on the role of the extracellular matrix in progressive retinal degenerative disorders. Invest Ophthalmol Vis Sci. 2013;54(13):8119–24. 24346621 10.1167/iovs.13-13536 PMC4587794

[7] Nishina S, Hirakata A, Hida T, Sawa H, Azuma N. CD44 expression in the developing human retina. Graefes Arch Clin Exp Ophthalmol. 1997;235(2):92–6. 9147957 10.1007/BF00941736

[8] Shinoe T, Kuribayashi H, Saya H, Seiki M, Aburatani H, Watanabe S. Identification of CD44 as a cell surface marker for Müller glia precursor cells. J Neurochem. 2010;115(6):1633–42. 20969572 10.1111/j.1471-4159.2010.07072.x

[9] Too LK, Gracie G, Hasic E, Iwakura JH, Cherepanoff S. Adult human retinal Müller glia display distinct peripheral and macular expression of CD117 and CD44 stem cell-associated proteins. Acta Histochem. 2017;119(2):142–9. 28110937 10.1016/j.acthis.2016.12.003

[10] Sneath RJ, Mangham DC. The normal structure and function of CD44 and its role in neoplasia. Mol Pathol. 1998;51(4):191–200. 9893744 10.1136/mp.51.4.191 PMC395635

[11] Zöller M. CD44, Hyaluronan, the hematopoietic stem cell, and leukemia-initiating cells. Front Immunol. 2015;6:235. 26074915 10.3389/fimmu.2015.00235 PMC4443741

[12] Graham VA, Marzo AL, Tough DF. A role for CD44 in T cell development and function during direct competition between CD44 + and CD44- cells. Eur J Immunol. 2007;37(4):925–34. 17330818 10.1002/eji.200635882

[13] Baaten BJ, Li C-R, Bradley LM. Multifaceted regulation of T cells by CD44. Commun Integr Biol. 2010;3(6):508–12. 21331226 10.4161/cib.3.6.13495 PMC3038050

[14] Protin U, Schweighoffer T, Jochum W, Hilberg F. CD44-deficient mice develop normally with changes in subpopulations and recirculation of lymphocyte subsets. J Immunol. 1999;163(9):4917–23. 10528194 10.4049/jimmunol.163.9.4917

[15] Ponta H, Sherman L, Herrlich PA. CD44: from adhesion molecules to signalling regulators. Nat Rev Mol Cell Biol. 2003;4(1):33–45. 12511867 10.1038/nrm1004

[16] Müller S, Sindikubwabo F, Cañeque T, Lafon A, Versini A, Lombard B, et al. CD44 regulates epigenetic plasticity by mediating iron endocytosis. Nat Chem. 2020;12(10):929–38. 32747755 10.1038/s41557-020-0513-5 PMC7612580

[17] Krishnamoorthy R, Agarwal N, Chaitin MH. Upregulation of CD44 expression in the retina during the rds degeneration. Brain Res Mol Brain Res. 2000;77(1):125–30. 10814838 10.1016/S0169-328X(00)00035-8

[18] Chaitin MH, Ankrum MT, Wortham HS. Distribution of CD44 in the retina during development and the rds degeneration. Brain Res Dev Brain Res. 1996;94(1):92–8. 8816281 10.1016/0165-3806(96)00046-6

[19] Chaitin MH, Brun-Zinkernagel AM. Immunolocalization of CD44 in the dystrophic rat retina. Exp Eye Res. 1998;67(3):283–92. 9778409 10.1006/exer.1998.0510

[20] Blank T, Goldmann T, Koch M, Amann L, Schön C, Bonin M, et al. Early Microglia Activation precedes photoreceptor degeneration in a mouse model of CNGB1-Linked Retinitis Pigmentosa. Front Immunol. 2017;8:1930. 29354133 10.3389/fimmu.2017.01930 PMC5760536

[21] Verbakel SK, van Huet RAC, Boon CJF, Hollander AI den, Collin RWJ, Klaver CCW, et al. Non-syndromic retinitis pigmentosa. Prog Retin Eye Res. 2018;66:157–86. 29597005 10.1016/j.preteyeres.2018.03.005

[22] Ferrari S, Di Iorio E, Barbaro V, Ponzin D, Sorrentino FS, Parmeggiani F. Retinitis pigmentosa: genes and disease mechanisms. Curr Genomics. 2011;12(4):238–49. 22131869 10.2174/138920211795860107 PMC3131731

[23] Hurley JB, Lindsay KJ, Du J. Glucose, lactate, and shuttling of metabolites in vertebrate retinas. J Neurosci Res. 2015;93(7):1079–92. 25801286 10.1002/jnr.23583 PMC4720126

[24] Gao Z, Zhu Q, Zhang Y, Zhao Y, Cai L, Shields CB, et al. Reciprocal modulation between microglia and astrocyte in reactive gliosis following the CNS injury. Mol Neurobiol. 2013;48(3):690–701. 23613214 10.1007/s12035-013-8460-4 PMC4079114

[25] Wang M, Wong WT. Microglia-Müller cell interactions in the Retina. Adv Exp Med Biol. 2014;801:333–8. 24664715 10.1007/978-1-4614-3209-8_42 PMC4685688

[26] Díaz-Lezama N, Kajtna J, Wu J, Ayten M, Koch SF. Microglial and macroglial dynamics in a model of retinitis pigmentosa. Vis Res. 2023;210:108268. 37295269 10.1016/j.visres.2023.108268

[27] Jentzsch MC, Tsang SH, Koch SF. A New Preclinical Model of Retinitis Pigmentosa due to Pde6g Deficiency. Ophthalmol Sci. 2023;3(4):100332. 37363133 10.1016/j.xops.2023.100332 PMC10285708

[28] Graca AB, Hippert C, Pearson RA. Müller glia reactivity and development of Gliosis in response to pathological conditions. Adv Exp Med Biol. 2018;1074:303–8. 29721957 10.1007/978-3-319-75402-4_37

[29] Kajtna J, Tsang SH, Koch SF. Late-stage rescue of visually guided behavior in the context of a significantly remodeled retinitis pigmentosa mouse model. Cell Mol Life Sci. 2022;79(3):148. 35195763 10.1007/s00018-022-04161-0 PMC8866266

[30] Lewis GP, Fisher SK. Up-regulation of glial fibrillary acidic protein in response to retinal injury: its potential role in glial remodeling and a comparison to vimentin expression. Int Rev Cytol. 2003;230:263–90. 14692684 10.1016/S0074-7696(03)30005-1

[31] Chaitin MH, Wortham HS, Brun-Zinkernagel AM. Immunocytochemical localization of CD44 in the mouse retina. Exp Eye Res. 1994;58(3):359–65. 7513650 10.1006/exer.1994.1026

[32] Bell OH, Copland DA, Ward A, Nicholson LB, Lange CAK, Chu CJ, et al. Single Eye mRNA-Seq reveals normalisation of the Retinal Microglial Transcriptome following acute inflammation. Front Immunol. 2019;10:3033. 31993055 10.3389/fimmu.2019.03033 PMC6964706

[33] Hoang T, Wang J, Boyd P, Wang F, Santiago C, Jiang L et al. Gene regulatory networks controlling vertebrate retinal regeneration. Science 2020; 370(6519). 10.1126/science.abb8598 PMC7899183 33004674

[34] Davis RJ, Tosi J, Janisch KM, Kasanuki JM, Wang N-K, Kong J, et al. Functional rescue of degenerating photoreceptors in mice homozygous for a hypomorphic cGMP phosphodiesterase 6 b allele (Pde6bH620Q). Invest Ophthalmol Vis Sci. 2008;49(11):5067–76. 18658088 10.1167/iovs.07-1422 PMC2715364

[35] Sakami S, Maeda T, Bereta G, Okano K, Golczak M, Sumaroka A, et al. Probing mechanisms of photoreceptor degeneration in a new mouse model of the common form of autosomal dominant retinitis pigmentosa due to P23H opsin mutations. J Biol Chem. 2011;286(12):10551–67. 21224384 10.1074/jbc.M110.209759 PMC3060508

[36] Claes E, Seeliger M, Michalakis S, Biel M, Humphries P, Haverkamp S. Morphological characterization of the retina of the CNGA3(-/-)Rho(-/-) mutant mouse lacking functional cones and rods. Invest Ophthalmol Vis Sci. 2004;45(6):2039–48. 15161873 10.1167/iovs.03-0741

[37] Laprell L, Tochitsky I, Kaur K, Manookin MB, Stein M, Barber DM, et al. Photopharmacological control of bipolar cells restores visual function in blind mice. J Clin Invest. 2017;127(7):2598–611. 28581442 10.1172/JCI92156 PMC5490774

[38] Jiang D, Liang J, Noble PW. Hyaluronan in tissue injury and repair. Annu Rev Cell Dev Biol. 2007;23:435–61. 17506690 10.1146/annurev.cellbio.23.090506.123337

[39] Young RW. Cell death during differentiation of the retina in the mouse. J Comp Neurol. 1984; 229(3):362–73. Available from: URL: https://pubmed.ncbi.nlm.nih.gov/6501608/. 10.1002/cne.902290307 6501608

[40] Swaroop A, Kim D, Forrest D. Transcriptional regulation of photoreceptor development and homeostasis in the mammalian retina. Nat Rev Neurosci. 2010;11(8):563–76. 20648062 10.1038/nrn2880 PMC11346175

[41] Jones BW, Pfeiffer RL, Ferrell WD, Watt CB, Marmor M, Marc RE. Retinal remodeling in human retinitis pigmentosa. Exp Eye Res. 2016;150:149–65. 27020758 10.1016/j.exer.2016.03.018 PMC5031517

[42] Haverkamp S, Wässle H. Immunocytochemical analysis of the mouse retina. J Comp Neurol. 2000;424(1):1–23. 10888735 10.1002/1096-9861(20000814)424:1<1::AID-CNE1>3.0.CO;2-V

[43] Fiorentino A, Sharp SI, McQuillin A. Association of rare variation in the glutamate receptor gene SLC1A2 with susceptibility to bipolar disorder and schizophrenia. Eur J Hum Genet. 2015;23(9):1200–6. 25406999 10.1038/ejhg.2014.261 PMC4351899

[44] Herishanu Y, Gibellini F, Njuguna N, Hazan-Halevy I, Keyvanfar K, Lee E, et al. CD44 signaling via PI3K/AKT and MAPK/ERK pathways protects CLL cells from spontaneous and drug induced apoptosis through MCL-1. Leuk Lymphoma. 2011;52(9):1758–69. 21649540 10.3109/10428194.2011.569962 PMC3403533

[45] Harada T, Harada C, Watanabe M, Inoue Y, Sakagawa T, Nakayama N, et al. Functions of the two glutamate transporters GLAST and GLT-1 in the retina. Proc Natl Acad Sci U S A. 1998;95(8):4663–6. 9539795 10.1073/pnas.95.8.4663 PMC22547

[46] Ishikawa M. Abnormalities in glutamate metabolism and excitotoxicity in the retinal diseases. Scientifica (Cairo). 2013; 2013:528940. 10.1155/2013/528940 PMC3872404 24386591

[47] Tabel M, Wolf A, Szczepan M, Xu H, Jägle H, Moehle C, et al. Genetic targeting or pharmacological inhibition of galectin-3 dampens microglia reactivity and delays retinal degeneration. J Neuroinflammation. 2022;19(1):229. 36115971 10.1186/s12974-022-02589-6 PMC9482176

[48] Ortega JT, Jastrzebska B. Neuroinflammation as a therapeutic target in Retinitis Pigmentosa and Quercetin as its potential modulator. Pharmaceutics 2021; 13(11). 10.3390/pharmaceutics13111935 PMC8623264 34834350

[49] Imai Y, Ibata I, Ito D, Ohsawa K, Kohsaka S. A novel gene iba1 in the major histocompatibility complex class III region encoding an EF hand protein expressed in a monocytic lineage. Biochem Biophys Res Commun. 1996;224(3):855–62. 8713135 10.1006/bbrc.1996.1112

[50] Paolicelli RC, Sierra A, Stevens B, Tremblay M-E, Aguzzi A, Ajami B, et al. Microglia states and nomenclature: a field at its crossroads. Neuron. 2022;110(21):3458–83. 36327895 10.1016/j.neuron.2022.10.020 PMC9999291

[51] Waller R, Baxter L, Fillingham DJ, Coelho S, Pozo JM, Mozumder M, et al. Iba-1-/CD68 + microglia are a prominent feature of age-associated deep subcortical white matter lesions. PLoS ONE. 2019;14(1):e0210888. 30682074 10.1371/journal.pone.0210888 PMC6347230

[52] Zhao L, Hou C, Yan N. Neuroinflammation in retinitis pigmentosa: therapies targeting the innate immune system. Front Immunol. 2022;13:1059947. 36389729 10.3389/fimmu.2022.1059947 PMC9647059

[53] Liu T, Zhang L, Joo D, Sun S-C. NF-κB signaling in inflammation. Signal Transduct Target Ther. 2017;2:17023–. 29158945 10.1038/sigtrans.2017.23 PMC5661633

[54] Eastlake K, Banerjee PJ, Angbohang A, Charteris DG, Khaw PT, Limb GA. Müller glia as an important source of cytokines and inflammatory factors present in the gliotic retina during proliferative vitreoretinopathy. Glia. 2015;64(4):495–506. 26556395 10.1002/glia.22942 PMC4981913

[55] Lesley J, Hyman R, Kincade PW. CD44 and its interaction with extracellular matrix. Adv Immunol. 1993;54:271–335. 8379464 10.1016/S0065-2776(08)60537-4

[56] Hubmacher D, Apte SS. The biology of the extracellular matrix: novel insights. Curr Opin Rheumatol. 2013;25(1):65–70. 23143224 10.1097/BOR.0b013e32835b137b PMC3560377

[57] Pintér P, Alpár A. The role of Extracellular Matrix in Human Neurodegenerative diseases. Int J Mol Sci 2022; 23(19). 10.3390/ijms231911085 PMC9569603 36232390

[58] Nedvetzki S, Gonen E, Assayag N, Reich R, Williams RO, Thurmond RL, et al. RHAMM, a receptor for hyaluronan-mediated motility, compensates for CD44 in inflamed CD44-knockout mice: a different interpretation of redundancy. Proc Natl Acad Sci U S A. 2004;101(52):18081–6. 15596723 10.1073/pnas.0407378102 PMC539795

[59] Izumi Y, Shimamoto K, Benz AM, Hammerman SB, Olney JW, Zorumski CF. Glutamate transporters and retinal excitotoxicity. Glia. 2002;39(1):58–68. 12112376 10.1002/glia.10082

[60] Rodríguez Villanueva J, Martín Esteban J, Rodríguez Villanueva LJ. Retinal cell Protection in Ocular Excitotoxicity diseases. Possible Alternatives offered by Microparticulate Drug Delivery systems and Future prospects. Pharmaceutics 2020; 12(2). 10.3390/pharmaceutics12020094 PMC7076407 31991667

[61] Vorwerk CK, Naskar R, Schuettauf F, Quinto K, Zurakowski D, Gochenauer G, et al. Depression of retinal glutamate transporter function leads to elevated intravitreal glutamate levels and ganglion cell death. Invest Ophthalmol Vis Sci. 2000;41(11):3615–21. 11006260

[62] Pfeiffer RL, Marc RE, Jones BW. Müller Cell metabolic signatures: evolutionary conservation and disruption in Disease. Trends Endocrinol Metab. 2020;31(4):320–9. 32187524 10.1016/j.tem.2020.01.005 PMC7188339

[63] Boccuni I, Fairless R. Retinal glutamate neurotransmission: from physiology to pathophysiological mechanisms of retinal ganglion cell degeneration. Life (Basel) 2022; 12(5). 10.3390/life12050638 PMC9147752 35629305

[64] Rauen T, Wiessner M. Fine tuning of glutamate uptake and degradation in glial cells: common transcriptional regulation of GLAST1 and GS. Neurochem Int. 2000;37(2–3):179–89. 10812203 10.1016/S0197-0186(00)00021-8

[65] Solier S, Müller S, Cañeque T, Versini A, Mansart A, Sindikubwabo F, et al. A druggable copper-signalling pathway that drives inflammation. Nature. 2023;617(7960):386–94. 37100912 10.1038/s41586-023-06017-4 PMC10131557

[66] Puré E, Cuff CA. A crucial role for CD44 in inflammation. Trends Mol Med. 2001;7(5):213–21. 11325633 10.1016/S1471-4914(01)01963-3

[67] Johnson P, Ruffell B. CD44 and its role in inflammation and inflammatory diseases. Inflamm Allergy Drug Targets. 2009;8(3):208–20. 19601881 10.2174/187152809788680994

[68] Khan AI, Kerfoot SM, Heit B, Liu L, Andonegui G, Ruffell B, et al. Role of CD44 and hyaluronan in neutrophil recruitment. J Immunol. 2004;173(12):7594–601. 15585887 10.4049/jimmunol.173.12.7594

[69] Cuff CA, Kothapalli D, Azonobi I, Chun S, Zhang Y, Belkin R, et al. The adhesion receptor CD44 promotes atherosclerosis by mediating inflammatory cell recruitment and vascular cell activation. J Clin Invest. 2001;108(7):1031–40. 11581304 10.1172/JCI200112455 PMC200948

[70] Jordan AR, Racine RR, Hennig MJP, Lokeshwar VB. The role of CD44 in Disease Pathophysiology and targeted treatment. Front Immunol. 2015;6:182. 25954275 10.3389/fimmu.2015.00182 PMC4404944

[71] Wang Y, Li L, Wu Y, Zhang S, Ju Q, Yang Y, et al. CD44 deficiency represses neuroinflammation and rescues dopaminergic neurons in a mouse model of Parkinson’s disease. Pharmacol Res. 2022;177:106133. 35182746 10.1016/j.phrs.2022.106133

[72] Wang Q, Teder P, Judd NP, Noble PW, Doerschuk CM. CD44 deficiency leads to enhanced neutrophil migration and lung injury in Escherichia coli pneumonia in mice. Am J Pathol. 2002;161(6):2219–28. 12466136 10.1016/S0002-9440(10)64498-7 PMC1850923

[73] Vohra R, Tsai JC, Kolko M. The role of inflammation in the pathogenesis of glaucoma. Surv Ophthalmol. 2013;58(4):311–20. 23768921 10.1016/j.survophthal.2012.08.010

[74] Re-engineering of. The damaged brain and spinal cord [electronic resource]: evidence-based neurorehabilitation. Springer.

[75] Haroon E, Miller AH, Sanacora G. Inflammation, glutamate, and Glia: a Trio of trouble in Mood disorders. Neuropsychopharmacology. 2017;42(1):193–215. 27629368 10.1038/npp.2016.199 PMC5143501

[76] Fogal B, Hewett SJ. Interleukin-1beta: a bridge between inflammation and excitotoxicity? J Neurochem. 2008;106(1):1–23. 18315560 10.1111/j.1471-4159.2008.05315.x

[77] Davis RJ, Hsu C-W, Tsai Y-T, Wert KJ, Sancho-Pelluz J, Lin C-S, et al. Therapeutic margins in a novel preclinical model of retinitis pigmentosa. J Neurosci. 2013;33(33):13475–83. 23946405 10.1523/JNEUROSCI.0419-13.2013 PMC3742933

[78] Grosche A, Hauser A, Lepper MF, Mayo R, von Toerne C, Merl-Pham J, et al. The proteome of native adult Müller glial cells from Murine Retina. Mol Cell Proteom. 2016;15(2):462–80. 10.1074/mcp.M115.052183 PMC4739667 26324419

[79] Wiśniewski JR, Zougman A, Nagaraj N, Mann M. Universal sample preparation method for proteome analysis. Nat Methods. 2009;6(5):359–62. 19377485 10.1038/nmeth.1322

