# Supplementary figures and images for: CD44 signaling in Müller cells impacts photoreceptor function and survival in healthy and diseased retinas
Source: J Neuroinflammation. 2024 Aug 2;21:190. doi: 10.1186/s12974-024-03175-8 (PMC11297696; doi:10.1186/s12974-024-03175-8)

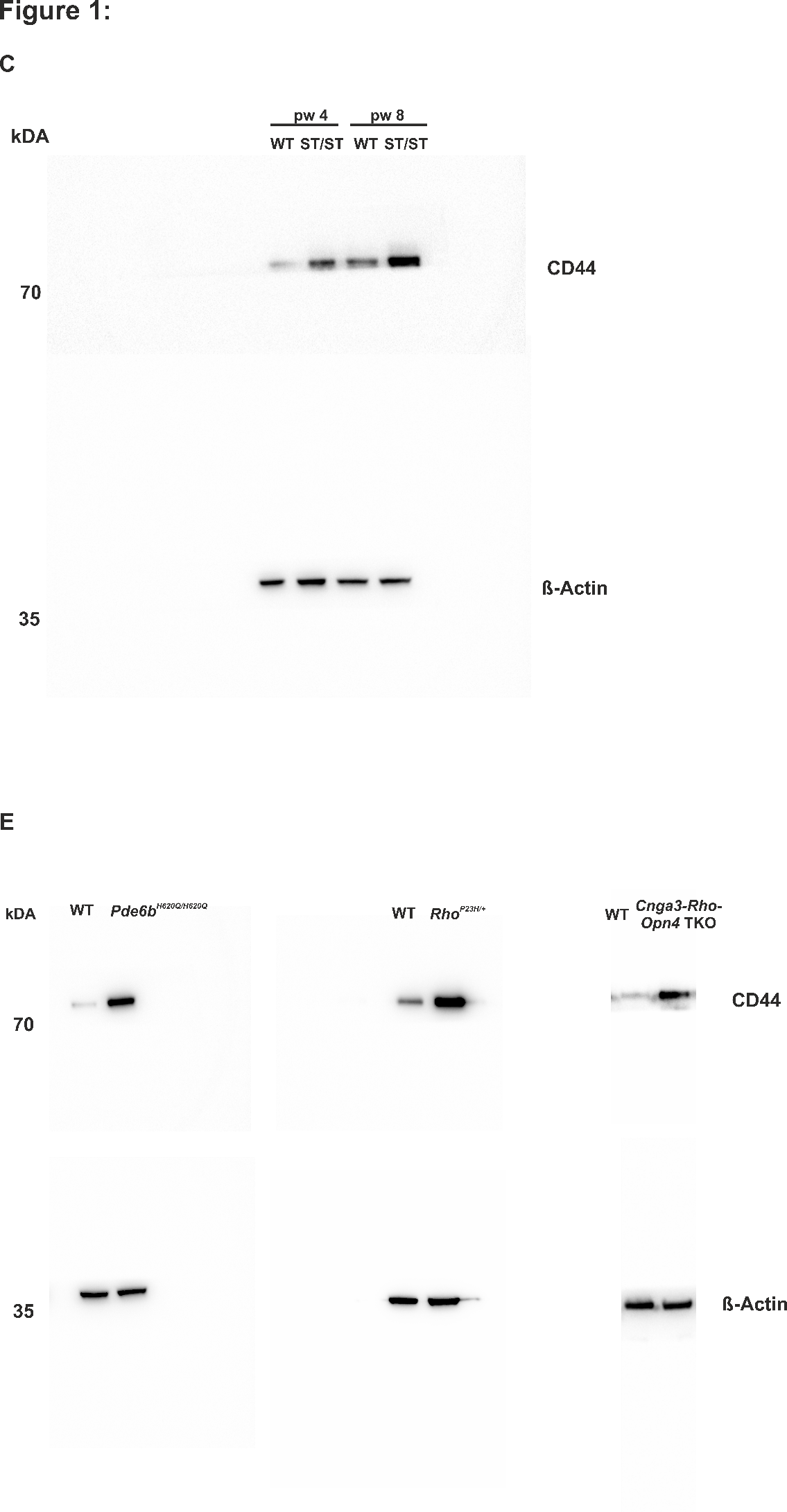


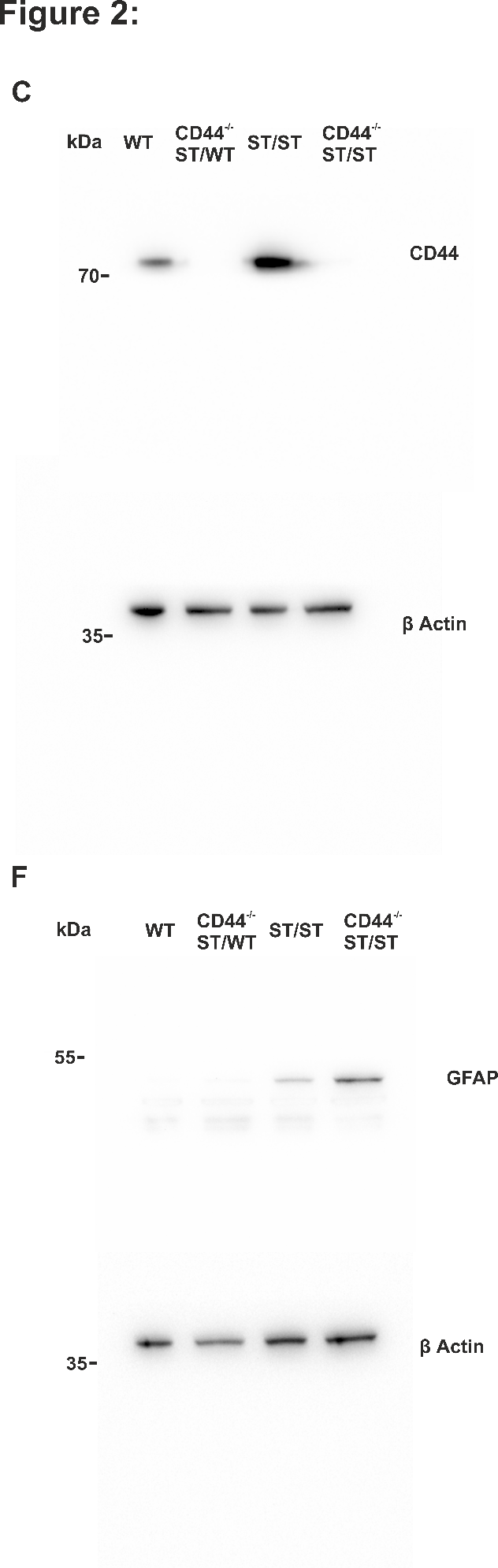


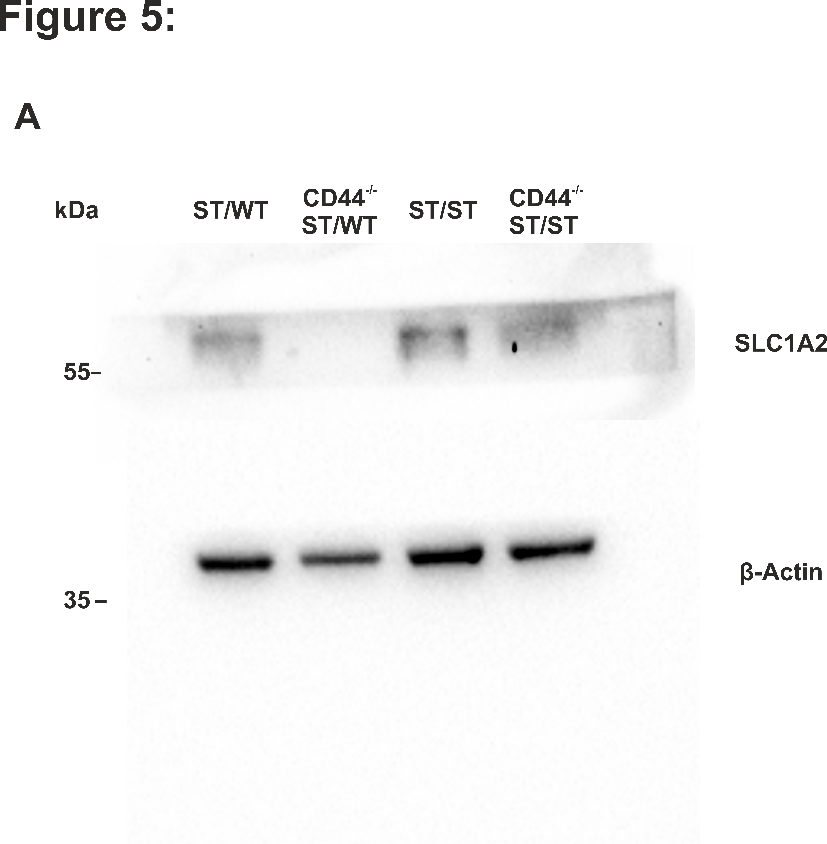

Supplement: Supplementary file 1 — Supplementary Material 1 [file 12974_2024_3175_MOESM1_ESM.docx]
