# Supplementary material for: CD44 signaling in Müller cells impacts photoreceptor function and survival in healthy and diseased retinas
Source: J Neuroinflammation. 2024 Aug 2;21:190. doi: 10.1186/s12974-024-03175-8 (PMC11297696; doi:10.1186/s12974-024-03175-8)
Supplement: Supplementary file 2 — Supplementary Material 2 [file 12974_2024_3175_MOESM2_ESM.docx]

**Supplemental Information**


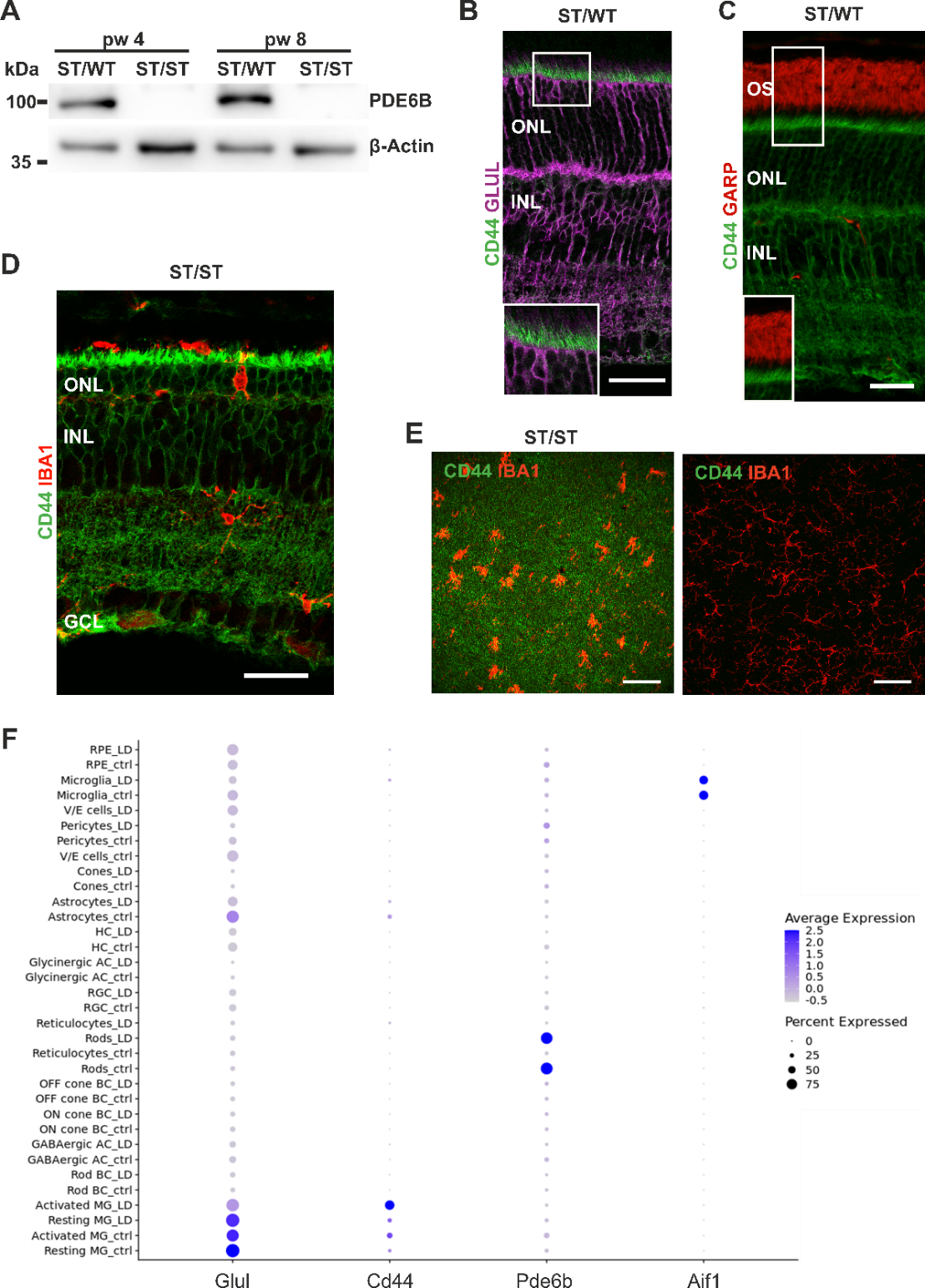
**Fig. S1.**

**Figure S1: PDE6B and CD44 expression in retinas.** **(A)** Retinas from ST/WT and ST/ST mice were analyzed at 4 and 8 weeks of age. Representative PDE6B immunoblot of retinal lysates. PDE6B was not detected in homozygous ST/ST retinas. β-Actin was used as a loading control. **(B-C)** Retinas from ST/WT mice were analyzed at 8 weeks of age. **(B)** Representative images of retinal sections immunostained for GLUL to visualize Müller cells and CD44. **(C)** Representative images of retinal sections immunostained for GARP to visualize rod photoreceptor outer segments (OS) and CD44. **(B-C)** Scale bar, 30 µm **(D-E)** Retinas from ST/ST mice were analyzed at 12 weeks of age. **(D)** Representative images of retinal sections immunostained for IBA1 to visualize microglia/macrophages and CD44. **(E)** Representative images of retinal flatmounts immunostained for IBA1 and CD44. Left panel, Z-stacks contain the complete depth of Müller cell microvilli. Right panel, Z-stacks contain the complete depth of the outer plexiform layer. Scale bar, 60 µm. **(F)** Analysis of scRNAseq data from (33) for Glul (Müller cell marker), Cd44, Pde6b (rod marker), and Aif1 (microglia marker) in WT (ctrl) and light-damaged (LD) retinas. AC, Amacrine cells; BC, Bipolar cells; HC, Horizontal cells; MG, Müller glia; RGC, Retinal Ganglion cells; RPE, retinal pigment epithelium; V/E cell, vascular/endothelial cells.


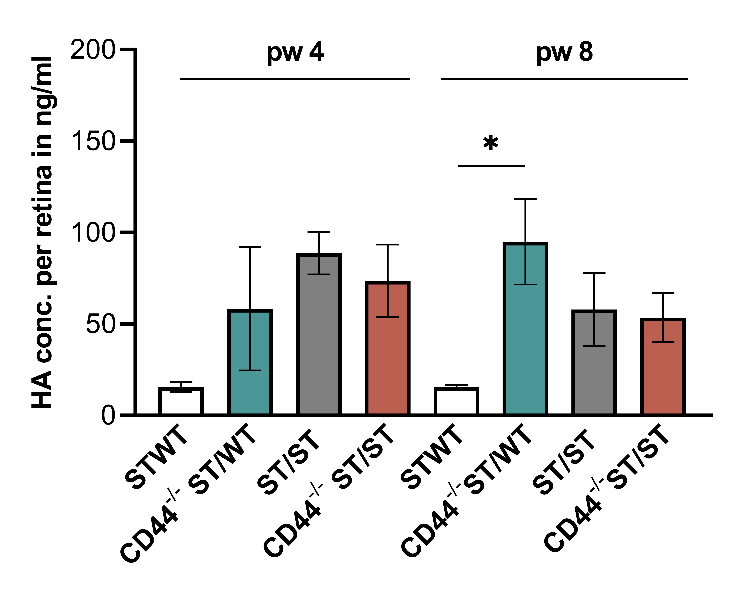
**Fig. S2.**

**Figure S2: Increased hyaluronic acid (HA) levels in CD44^−/−^ retinas.** Quantitative measurements of HA in retinas from Pde6b^STOP/WT^ (ST/WT), Cd44^-/-^ Pde6b^STOP/WT^ (CD44^-/-^ ST/WT), Pde6b^STOP/STOP^ (ST/ST), and Cd44^‑/‑^ Pde6b^STOP/STOP^ (CD44^‑/‑^ ST/ST) mice at 4 and 8 weeks of age using ELISA. pw 4, N = 3 for ST/WT, CD44^‑/‑^ ST/WT, and ST/ST, N = 5 for CD44^‑/‑^ ST/ST. pw 8, N = 5 for ST/WT, CD44^‑/‑^ ST/WT, and CD44^‑/‑^ ST/ST, N = 6 for ST/ST. Data, presented as mean ± SEM, were compared by ANOVA. * P ≤ .05.

**Fig. S3.**


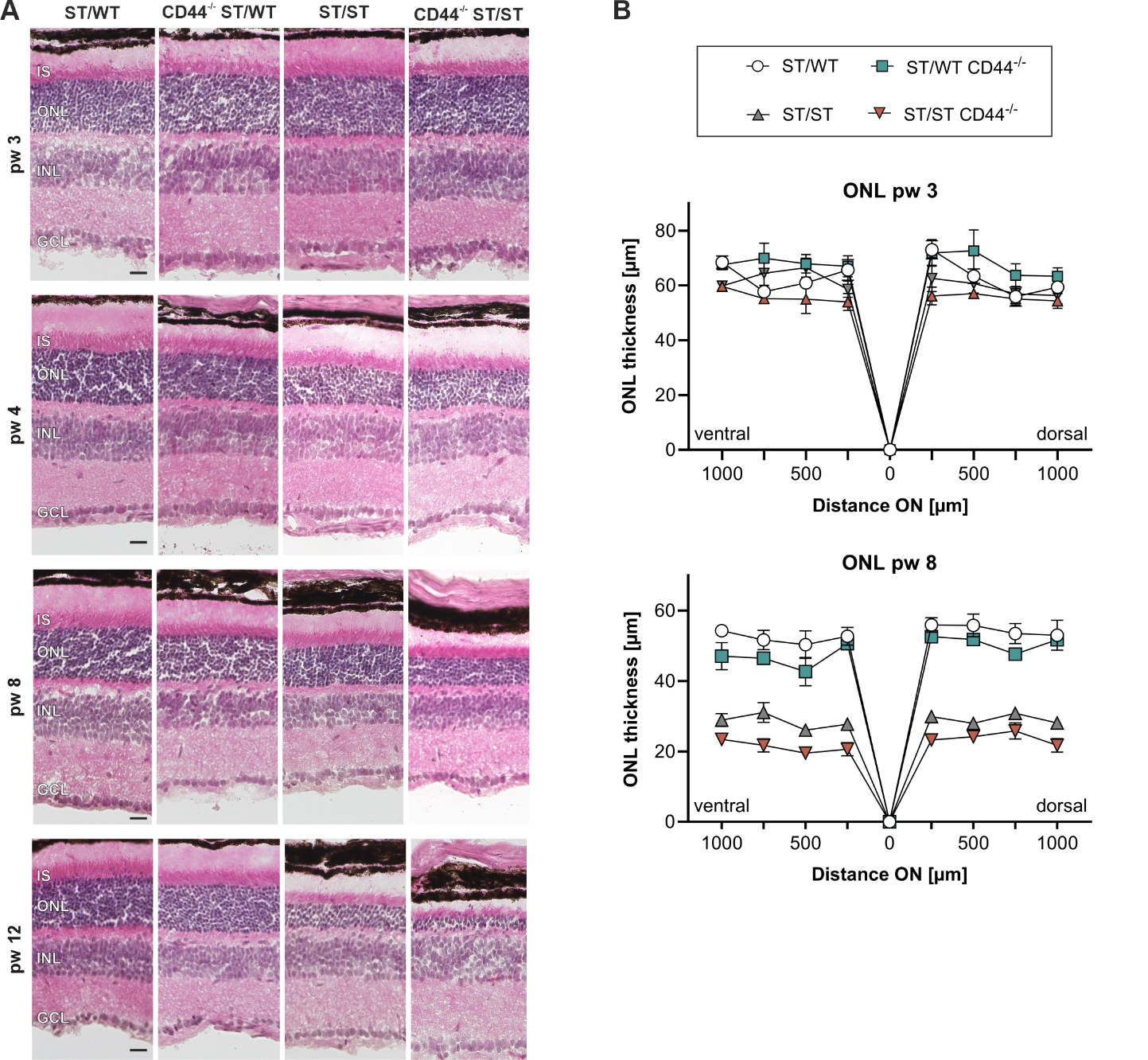


Fig. S3: Loss of CD44 did not affect retinal development but led to increased photoreceptor degeneration. (A) Retinal sections from ST/WT, CD44^-/-^ ST/WT, ST/ST, and CD44^-/-^ ST/ST mice were analyzed at 3,4, 8, and 12 weeks of age using hematoxylin and eosin staining. Scale bar, 15 µm. IS, inner segments; ONL, outer nuclear layer; INL inner nuclear layer; GCL, ganglion cell layer. (B) ONL thickness of retinal sections from ST/WT, CD44^-/-^ ST/WT, ST/ST, and CD44^-/-^ ST/ST mice at 3 and 8 weeks of age through the optic nerve (ON) at 3 (upper panel) and 8 weeks of age (lower panel). *N* = 3 per group. Data represents mean ± SEM.

**Fig. S4.**


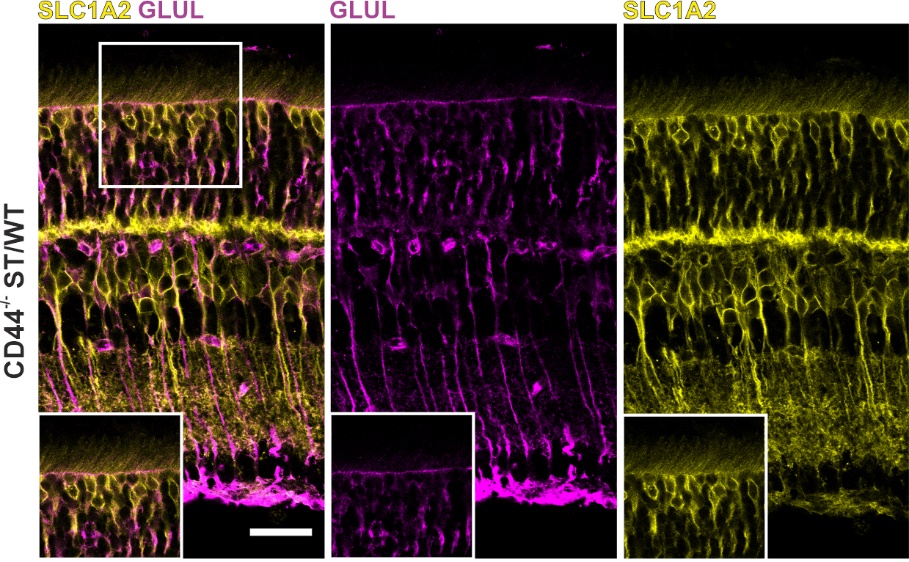


**Fig. S4: Glutamate transporter SLC1A2 is expressed in Müller cells.** Representative images of retinal sections from 12-week-old CD44^-/-^ ST/WT mice immunostained for glutamine synthetase (GLUL) to visualize Müller cells and for SLC1A2. Scale bar, 25 µm.
